# Quantitative real-time PCR analysis of *Anopheles dirus TEP1* and *NOS* during *Plasmodium berghei* infection, using three reference genes

**DOI:** 10.7717/peerj.3577

**Published:** 2017-07-26

**Authors:** Jonathan W.K. Liew, Mun Yik Fong, Yee Ling Lau

**Affiliations:** Department of Parasitology, Faculty of Medicine, University of Malaya, Kuala Lumpur, Malaysia

**Keywords:** *Anopheles dirus*, *Plasmodium berghei*, TEP1, NOS, Reference genes, Normalization

## Abstract

Quantitative reverse transcription PCR (qRT-PCR) has been an integral part of characterizing the immunity of *Anopheles* mosquitoes towards *Plasmodium* invasion. Two anti-*Plasmodium* factors of *Anopheles*, thioester-containing protein 1 (TEP1) and nitric oxide synthase (NOS), play a role in the refractoriness of *Anopheles* towards *Plasmodium* infection and are generally expressed during infection. However, these are less studied in *Anopheles dirus*, a dominant malaria vector in Southeast Asia. Furthermore, most studies used a single reference gene for normalization during gene expression analysis without proper validation. This may lead to erroneous quantification of expression levels. Therefore, the present study characterized and investigated the expression profiles of *TEP1* and *NOS* of *Anopheles dirus* during *P. berghei* infection*.* Prior to that, the *elongation factor 1-alpha* (*EF1*), *actin 1* (*Act*) and *ribosomal protein S7* (*S7*) genes were validated for their suitability as a set of reference genes. *TEP1* and *NOS* expressions in *An. dirus* were found to be significantly induced after *P. berghei* infection.

## Introduction

Advances in molecular biology and gene expression techniques have enabled researchers to identify genes involved in the immunological response of *Anopheles* mosquitoes towards *Plasmodium* infection (Chen, Mathur & James, 2008; Volohonsky et al., 2017). Understanding of this immunity complex may lead to development of novel approaches for controlling malaria transmission, such as transgenic mosquitoes and inhibition of parasite development in the mosquito. The immunological changes caused by *Plasmodium* infection at the transcriptome level can be captured in microarray gene expression and quantitative reverse transcription PCR (qRT-PCR) analyses (Aguilar et al., 2005). The qRT-PCR is used as a tool to validate expression data from microarray analyses and to detect changes in the expression levels of target genes.

Thioester-containing protein 1 (TEP1) has been shown to mediate anti-*Plasmodium* responses in various mosquito species (Clayton, Dong & Dimopoulos, 2014; Jaramillo-Gutierrez et al., 2009). In the hemolymph of the mosquito, TEP1, leucine-rich repeat immune protein 1 (LRIM1) and *Anopheles*-*Plasmodium*-responsive leucine-rich repeat 1 (APL1) protein combine to form a stable complex (Fraiture et al., 2009; Povelones et al., 2011). The precise mode of action of this TEP1-associated complex is not known but it has been implicated in the vector competence of *An. gambiae* (Blandin et al., 2004; Fraiture et al., 2009) and *An. quadrinnulatus* (Habtewold et al., 2008). Silencing of the TEP1, LRIM1 or APL1 gene abolishes parasite melanization and converts refractory or non-compatible *Anopheles* strain into a susceptible one.

Nitric oxide synthase (NOS) catalyzes the conversion of L-arginine to L-citrulline in the mosquito, producing nitric oxide which is toxic to the parasite (Clayton, Dong & Dimopoulos, 2014). The generated nitric oxide in the midgut cells and subsequent tyrosine nitration kill the parasite ookinetes as they traverse the midgut epithelial cells (Kumar et al., 2004). Furthermore, NOS plays a role in hemocyte-mediated immune responses in the hemocoel of the mosquito and is expressed in the hemocytes and fat body (Hillyer & Estevez-Lao, 2010). Elevated levels of *NOS* gene expression have been observed in the midgut epithelial cells of *An. stephensi* invaded by *P. berghei* ookinetes (Han et al., 2000). Moreover, chemical inhibition of NOS resulted in an increase in the number of oocysts in infected *An. stephensi* (Luckhart et al., 1998) and *An. culicifacies* B (Vijay et al., 2011). These findings indicate that NOS is also a determinant of vector refractoriness in *Anopheles* mosquitoes (Vijay et al., 2011). Interestingly, a study has shown that some NOS activity is required for survival of the *Plasmodium* at the early developmental stages but it has an opposite effect at a later stage by limiting oocyst survival in the mosquito (Gupta et al., 2009).

The TEP1 and NOS may be working in concert to eliminate parasites in the mosquito. The heme peroxidase (HPX2)/NADPH oxidase 5 (NOX5) system of *An. gambiae*, along with NOS, mediates nitration of the midgut epithelial cells and potentiates nitric oxide toxicity against *P. berghei*. Evidence shows that epithelial nitration precedes TEP1-mediated lysis. De Almeida Oliveira, Lieberman & Barillas-Mury (2012) proposed that epithelial nitration is needed for effective TEP1-mediated lysis of ookinetes. Furthermore, silencing the TEP1, leucine-rich repeat proteins and HPX2/NOX5, renders the resistant *An. gambiae* L3-5 strain susceptible to *P. berghei* infection. These findings demonstrate the concerted roles of the proteins in the defense mechanism of *Anopheles* mosquitoes (Eldering et al., 2016).

It is evident that these studies have greatly advanced our understanding of *Anopheles* immunity and qRT-PCR has been featured as an important investigative tool in these informative studies. However, most of the investigations used only one reference gene (often, the *ribosomal protein S7* gene) for normalization of expression data and no validation was documented. As robust as qRT-PCR is, it may still be subjected to systematic error from technical and biological limitations (Kozera & Rapacz, 2013; Omondi et al., 2015). Thus, proper normalization is crucial to ensure an accurate result. It is recommended that at least three different reference genes be used for normalization as this will increase accuracy and resolution. Furthermore, the reference genes for normalization should be validated for different experimental designs to obtain reliable gene expression results (Bustin et al., 2009; Derveaux, Vandesompele & Hellemans, 2010; Ponton et al., 2011).

*Anopheles dirus* is the primary malaria vector in Southeast Asia and is capable of transmitting all human malaria parasites (Coatney et al., 1971; Manguin et al., 2008; Marchand et al., 2011; Nakazawa et al., 2009; Vythilingam et al., 2005). However, molecular understanding of immune responses in *An. dirus* is still lacking. The recent availability of the *An. dirus* genome sequence (Neafsey et al., 2015) should facilitate and enhance research on its immune response mechanisms. Given the important roles TEP1 and NOS play in the immune defense of *An. gambiae* and *An. stephensi* against *Plasmodium*, we postulate that both immune factors in *An. dirus* would play similar roles against the parasites.

Thus, the current study aims to have better understanding of the molecular immune responses in an important Southeast Asian malaria vector. The implementation of a proper qRT-PCR assay is also important to yield data that is more comparable and reliable. Indeed, these will advance the characterization of immune traits and transmission mechanisms of major malaria vectors in a local context. Aside from a few of studies reporting the use of the *An. dirus*/*P. berghei* model (Fracisco et al., 2010; Lapcharoen et al., 2012), *An. dirus* has been determined to be permissive to *P. berghei* infection in the laboratory (JWK Liew, 2017, unpublished data). Hence, the current study validated elongation factor 1-alpha (*EF1*), actin 1 (*Act*) and *S7* as reference genes for normalization in qRT-PCR, followed by investigation on *TEP1* and *NOS* expression profiles in *An. dirus* during *P. berghei* ANKA infection.

## Materials and Methods

### Total RNA extraction of whole mosquitoes and cDNA synthesis

Total RNA was extracted from pooled whole mosquitoes as previously described (Khan et al., 2016). Each batch of RNA was extracted from three female mosquitoes using ReliaPrep™ RNA Tissue Miniprep System (Promega, Madison, WI, USA). The pooled mosquitoes were first cold-anesthetized before homogenization in cold lysis buffer using the handheld homogenizer, BioMasher-II (Nippi, Tokyo, Japan). The total RNA was then rendered DNA-free using TURBO DNA-*free*™ kit (Ambion, Vilnius, Lithuania). cDNA was synthesized from 150 ng of total RNA using the qPCRBIO cDNA synthesis kit (PCR Biosystems, London, UK).

### PCR amplification of *AdTEP1* and *AdNOS* coding sequences and of *AdEF1* and *AdAct* partial sequences

At the time of this study, the *An. dirus* genome was not sequenced. Only *An. dirus TEP1* (GenBank: FJ263422) and *S7* (GenBank: AY369135.2) partial sequences were available. Thus, rapid amplification of cDNA ends (RACE), using the SMARTer™ RACE cDNA Amplification kit (Clontech, California, USA) was performed to obtain full length coding sequences of *AdTEP1* and *AdNOS*. Nested RACE PCR was carried out according to the manufacturer’s instructions. For elongation factor 1-alpha, *AdEF1* was amplified using degenerate primers designed based on aligned sequences of *Aedes aegypti* (GenBank: DQ440206), *Culex quinquefasciatus* (GenBank: XM_001850793) and *An. gambiae* (GenBank: XM_308429.3), while partial sequence of *AdAct* was amplified using universal primers designed by Staley et al. (2010). These PCR amplifications were performed using DreamTaq Green DNA polymerase (Thermo Fisher Scientific, Waltham, MA, USA). All amplicons were cloned into pGEM®-T vector (Promega, Madison, USA) and sequenced. The primers used for both PCR are shown in Table 1.

**Table 1:** Primers used in PCR.

Primer	Sequence (5′–3′)
*TEP1*	
GSP1: Ad5RaTEP1	GTCCTAGAACCCTGATGCTCCAGCAGTGC
NGSP1: Ad5RaTEP1N1	CGATGTCAGCGCTACACCATTCCGCAGACC
GSP2: Ad3RaTEP1	CAAGCAGACGGCTCCTTCGGTGTGTGG
NGSP2: Ad3RaTEP1N2	GCTGGTTGAGAGGGCATACGAGTGGCTCG
*NOS*	
GSP1: AdNOS5Ra	CCTCGCGCGACAGTGCGAGGAACACCCG
NGSP1: Ad5RaNOSN1	CTGGACCATCTCCTGCTTCTCGTCCCGG
GSP2: AdNOS3Ra	GTGCGCAGCGCACCGTCGTTCCACATGTCG
NGSP2: Ad3RaNOSN2	CCGACCAAGCCGGTCATCCTGATCGGTC
*EF1*	
EF1-F1	ATGGGTAAGGARAAGACTCA
EF1-R1	GACCTTCTCCTTGATYTCG
*Act*	
Act-2F	ATGGTCGGYATGGGNCAGAAGGACTC
Act-8R	GATTCCATACCCAGGAAGGADGG

### Molecular and phylogenetic analysis of *AdTEP1* and *AdNOS*

The *AdTEP1* and *AdNOS* consensus sequences were formed from the 5′-RACE and 3′-RACE PCR products of each gene using BioEdit Sequence Alignment Editor v7.2.3. BLAST (Johnson et al., 2008) and Open Reading Frame Finder (ORFfinder) programs from NCBI were used to obtain and analyze the coding sequences of *AdTEP1* and *AdNOS*. Putative conserved domains and protein structure were analyzed using BLASTP. The TEP1 and NOS sequences of several insects were aligned using Clustal Omega and phylogenetic trees were constructed (Jukes-Cantor model; neighbor-joining method; 1,000 bootstrap value) using MEGA 7.0.21 software. All sequences were submitted to GenBank.

### *Plasmodium berghei* infection of *An. dirus* mosquitoes

*Anopheles dirus* WRAIR2 strain was reared at a temperature of 25–27 °C, 70–80% humidity and 12:12 h light-dark photoperiod. In every experimental replicate, five- to seven-day old female *An. dirus* mosquitoes, emerged from the same larva tray were used. The mosquitoes were placed in the same cage and given 10% sugar solution supplemented with Vitamin B complex, *ad libitum*. One night before blood feeding on a *P. berghei* ANKA-infected BALB/c female mouse, female mosquitoes were starved in an ambient temperature of 20 ± 1 °C and 80% humidity. Subsequently, blood feeding and containment of engorged mosquitoes were performed in the same environmental conditions. Female mosquitoes fed on healthy mouse were used as controls. All mosquito infections were performed at gametocytemia of 0.42–0.64% (parasitemia: 5.86–6.36%). It was found that fully engorged mosquitoes which fed on such levels of parasites, will have a substantial number of oocysts in their midguts and are 100% prevalent with sporozoites present in their salivary glands(JWK Liew, 2017, unpublished data). Ethical approval for the study was obtained from the Faculty of Medicine Institutional Animal Care and Use Committee, University of Malaya, Malaysia (Ethics Reference no.: 20150407/PARA/R/MBK).

### Quantitative reverse transcription PCR of *AdTEP1* and *AdNOS* post *P. berghei* infection

Total RNA was extracted at 12 h, 24 h, 48 h and Day 5 post infection (PI) and reverse transcribed. The PCR conditions were the same for all primers (Table 2) i.e., 95 ° C for 30 s, 40 cycles of 95 °C for 10 s and 57.6 °C for 25 s; and a melt curve step from 65 to 95 °C, with increment of 0.5 °C every 5 s. The qPCR was performed using SsoAdvanced™ Universal SYBR® Green Supermix (Bio-Rad, Hercules, CA, USA) on Bio-Rad CFX96™ Real-Time System. The primers were designed using the online PrimerQuest tool on Integrated DNA Technologies website, following the suggestions in the SsoAdvanced™ Universal SYBR® Green Supermix instruction manual. A 20 µL reaction contained 312.5 nM of each forward and reverse primer and 6 ng of cDNA. Results of the PCR were analyzed by the Bio-Rad CFX Manager™ 3.1 software. Expression levels were normalized against those of *EF1*, *Act* and *S7*. Relative expression was calculated using the 2^−ΔΔ*C*^_*T*_ formula, compared to the controls. The experiment was repeated 4 times.

**Table 2:** Primers used in qRT-PCR.

Primer	Sequence (5′–3′)	Expected size (bp)	PCR efficiency
*TEP1*			
AdrtTEP1F3	GGCAAAGTCCATGCAAAC	126	103.3
AdrtTEP1R3	ATAACGGAACCAACCTCATC		
*NOS*			
AdrtNOSF2	GGAGAAAGCGCACATCTAC	116	109.2
AdrtNOSR2	ACTTCTCCATTTCCGTTTCC		
*EF1*			
AdrtEF1F1	CCGGACATCGTGATTTCAT	118	102.8
AdrtEF1R1	TGGCCGTTCTTGGAGATA		
*Act*			
AdrtACTF2	TCTGACCGACTACCTGAT	130	100.0
AdrtACTR2	CATCTCCTGCTCGAAGTC		
*S7*			
AdrtS7F1	GAGGTCGAGTTCAACAACAA	132	108.4
AdrtS7R1	GAACACGACGTGCTTACC		

### Gene expression stabilities and statistical analysis

To determine the gene expression stabilities of *AdEF1*, *AdAct* and *AdS7*, NormFinder v0.953 Microsoft Excel add-in (Andersen, Jensen & Orntoft, 2004) and Bio-Rad CFX Manager™ 3.1 software were used. The CFX manager software determines the target stability values by adopting the pairwise variation strategy as in geNorm (Vandesompele et al., 2002). Additionally, the comparative delta-Ct method (Silver et al., 2006) was also employed toestablish gene stability. For statistical analysis, one-way ANOVA with multiple comparisons of Tukey was performed with 95% confidence intervals (alpha = 0.05) using GraphPad Prism version 5.01.

## Results

### Molecular and phylogenetic analyses of *AdTEP1* and *AdNOS* proteins

**Figure 1:**
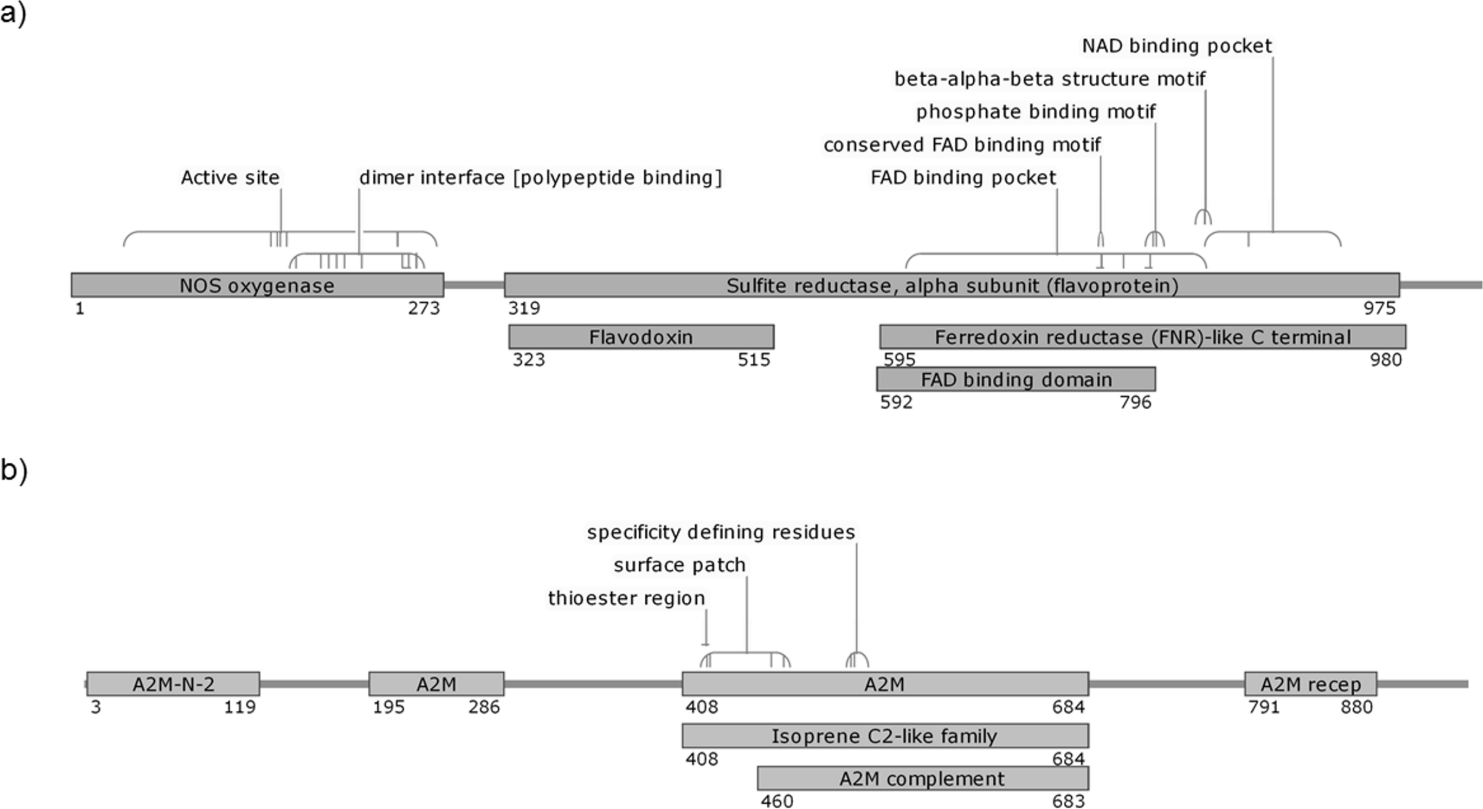
Putative conserved domains of *An. dirus* (A) NOS and (B) TEP1 protein detected by BLASTP. The numbers denote the region of the domains on the protein sequence. The diagrams are not drawn to scale. FAD, flavine adenine dinucleotide; NAD, nicotinamide adenine dinucleotide; A2M-N-2, Alpha-2-macroglobulin family N-terminal region; A2M, Protein similar to Alpha-2-macroglobulin; A2M recep, Alpha-2-macroglobulin receptor; A2M complement, Alpha-2-macroglobulin complement component.

Details of the proteins are shown in Table 3. The *AdTEP1* coding sequence has 71% and 98% similarity to *An. gambiae TEP-1* mRNA (GenBank: AF291654.1) and *An. dirus* strain WRAIR2 contig 1.4921, whole genome shotgun (WGS) sequence (GenBank: APCL01004922.1), respectively. Putative conserved domains detected on the protein include alpha-2 macroglobulin associated components (A2M-N-2, A2M receptor, A2M complement component), thioester regions and isoprene C2-like family (Fig. 1). As for *AdNOS*, it is shown to be 89% similar to *An. gambiae* str. PEST AGAP008255-PA mRNA (GenBank: XM_317213.1) and 99% similar to *An. dirus* strain WRAIR2 contig 1.1607, WGS sequence (GenBank: APCL01001608.1). BLASTP detected typical conserved domains of NOS proteins in the protein sequence (Fig. 1).

**Table 3:** Coding DNA region length and protein length of genes sequenced in this study.

Gene	Total base pairs (bp)	Protein length (amino acids)	GenBank accession no.	Isoelectric point^a^	Molecular weight (Dalton)^a^
*TEP1*	2,733	910	KY465474	6.91	102644.20
*NOS*	3,006	1,001	KY465473	6.22	114212.80
*EF1*	1,137	–	KY022437	–	–
*Act*	683	–	KY022436	–	–

**Notes.**

a Predicted by ExPASy Compute pI/Mw tool (Gasteiger et al., 2003).

**Figure 2:**
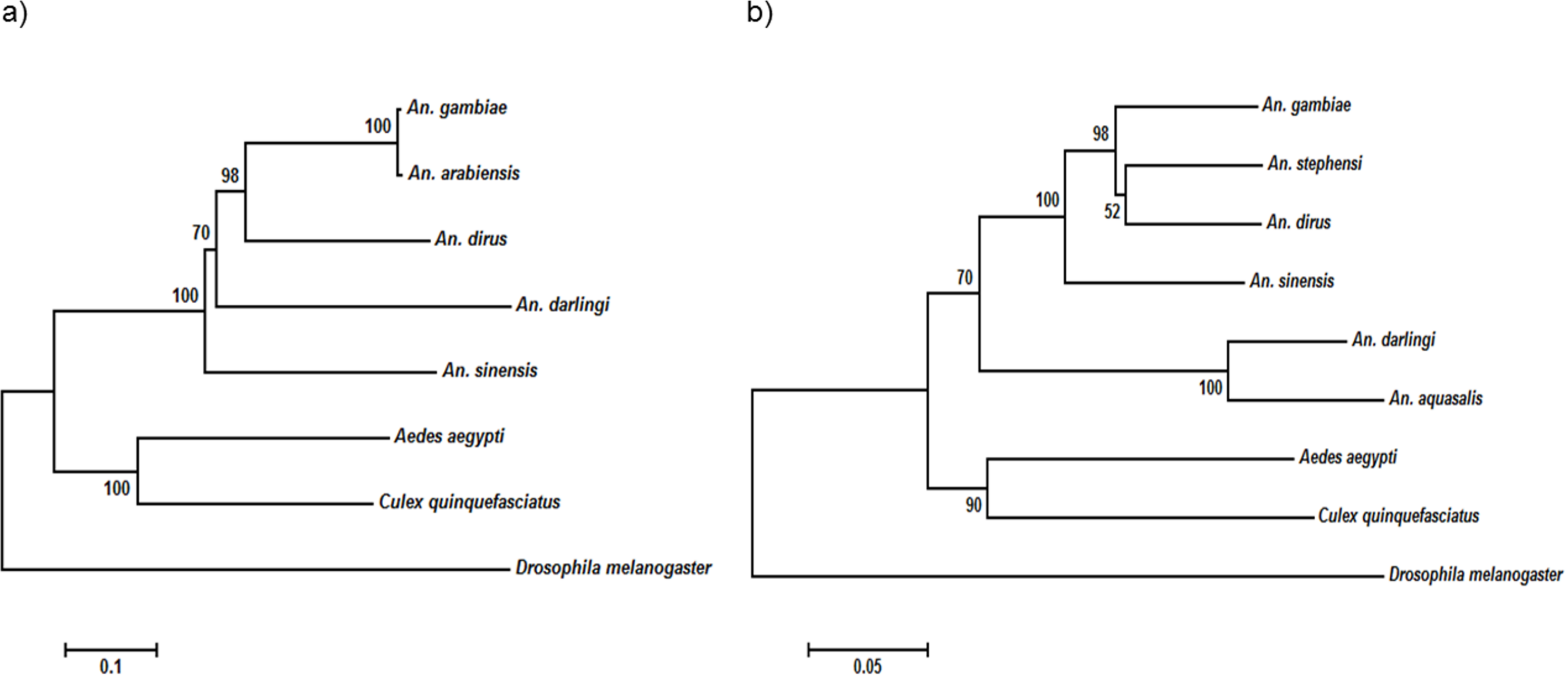
Phylogenetic tree of (A) *TEP1* gene and (B) *NOS* gene. The scale bar at the bottom are in the units of number of base substitutions per site.

The NOS protein is typically conserved among insect species, demonstrating high sequence similarity, especially among the anopheline mosquitoes. On the other hand, the insects’ TEP1 protein sequences are considerably conserved, but do not exhibit high sequence identity as that of NOS protein (Table 4). Multiple sequence alignment (File S1) showed that the TEP1 protein sequences vary among the insects, albeit are more similar within the *Anopheles* genus. *AdTEP1* is closer in homology to that of *An. gambiae* and *An. arabiensis*. This is also reflected in the phylogenetic tree (Fig. 2).

**Table 4:** Percentage similarity of *AdTEP1* and *AdNOS* proteins to those of other organisms, calculated by Clustal Omega, available at the EMBL-EBI website (Sievers et al., 2011).

Organism	GenBank accession no.	% Identity
TEP1		
*An. gambiae*	AAG00600	72.28
*An. arabiensis*	ACG68535	72.28
*An. sinensis*	KFB36250	61.67
* An. darlingi*	ETN59728	54.71
*Ae. aegypti*	XP_001660377	42.54
*Cx. quinquefasciatus*	XP_001842016	41.48
* D. melanogaster*	NP_523578	28.17
NOS		
*An. stephensi*	O61608.2	97.60
* An. gambiae*	XP_317213	96.20
* An. sinensis*	KFB45232	95.10
* An. darlingi*	ETN60994	85.47
*An. aquasalis*	AEK26396	78.21
* Ae. aegypti*	XP_001660328	88.50
*Cx. quinquefasciatus*	XP_001842036	75.88
* D. melanogaster*	NP_001027243.2	73.75

### *EF1*, *Act* and *S7* are suitable reference genes for the study

The suitability of *EF1*, *Act* and *S7* as reference genes for gene expression analysis of *P. berghei*-infected *An. dirus* was addressed under the experimental conditions of this study, in conjunction with the SYBR green intercalating dye chemistry. The stability values of the three genes, as analyzed by the Bio-Rad CFX Manager software showed that they were stably expressed as the values were in the acceptable range. When all three genes were collectively used across 16 separate qRT-PCR reactions (4 time points × 4 replicates), the average mean coefficient of variance (CV) was 0.109 (SD: 0.053) while the average mean*M* value was 0.271 (SD: 0.133). Stably expressed reference genes of homogeneous samples should exhibit a mean CV of <0.25 and expression stability, *M* value of <0.5 (Hellemans et al., 2007).

**Figure 3:**
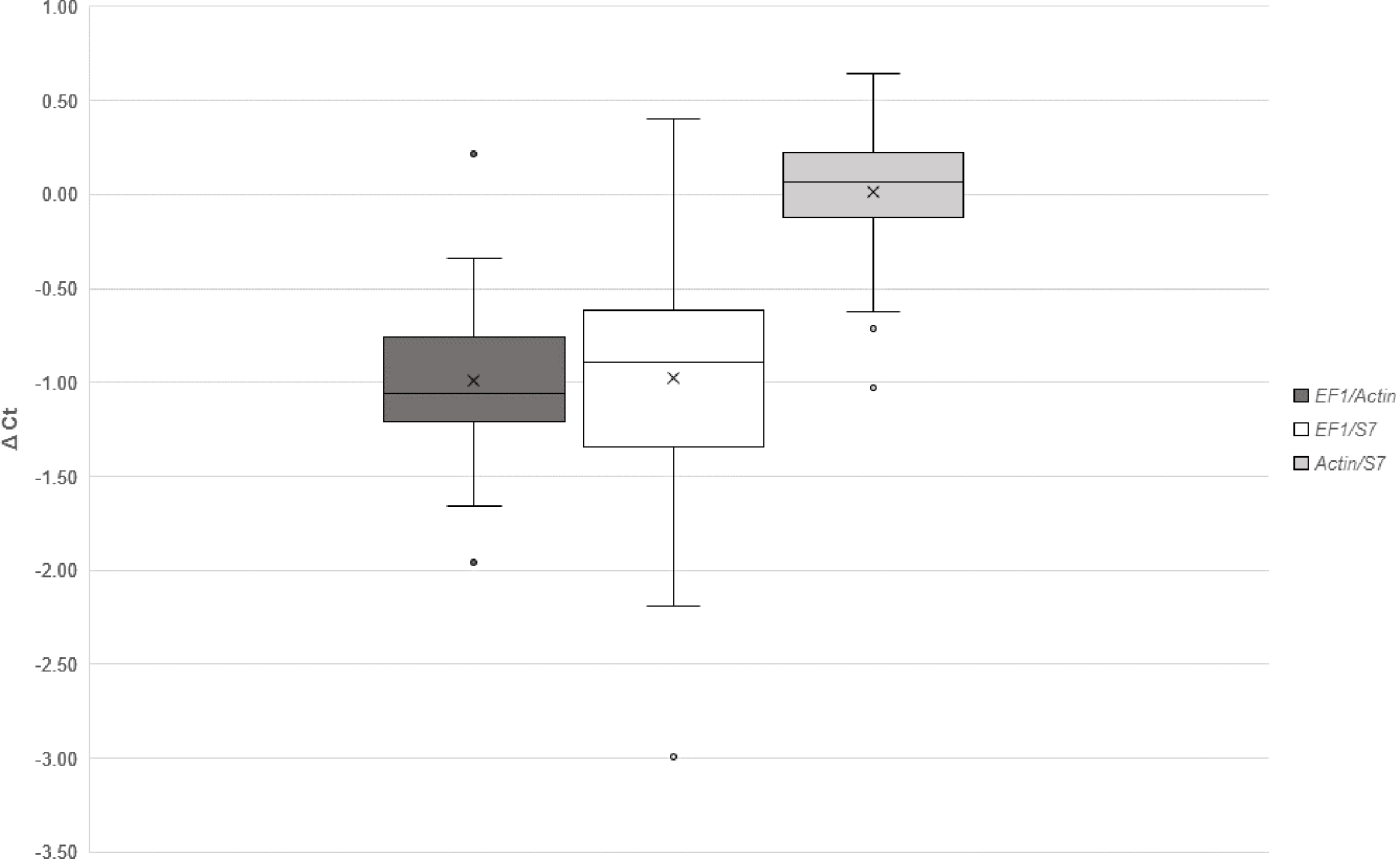
Comparative Δ*Ct* method for selection of reference genes.

NormFinder identified *Act* as the best reference gene and *Act*/*S7* (stability value: 0.040) as the best combination when comparing normal blood fed and infected groups. Whereas, in experiments comparing between timepoints, *Act* was also the best gene but the best combination of two genes was *EF1*/*Act* (stability value: 0.095). Using the comparative delta-Ct method, the best combinations in descending order were *Act*/*S7*, *EF1*/*Act*, *EF1*/*S7* (Fig. 3). geNORM also identified combination *Act*/*S7* (*M* value: 0.023) as the best, followed by *EF1*/*Act* (*M* value: 0.028) and *EF1*/*S7* (*M* value: 0.041). Furthermore, the relative expressions of *AdTEP1* and *AdNOS* were compared when a single or combination of reference genes were used for normalization (Table 5). Variation could be seen when expression was normalized against a single reference gene. Nevertheless, to adhere to the MIQE guidelines (Bustin et al., 2009), these three reference genes were used together in the current gene expression study.

**Table 5:** Normalized relative expressions of *AdTEP1* and *AdNOS* at 24 h and 48 h PI using different combination of reference gene(s).

Gene	Reference gene	24 h PI	48 h PI
*TEP1*	*EF1*	3.533	1.091
	*Act*	2.685	1.499
	*S7*	3.187	1.435
	*Act*/*S7*	2.925	1.467
	*EF1*/*Act*	3.08	1.278
	*EF1*/*S7*	3.356	1.251
	*EF1*/*Act*/*S7*	3.115	1.329
*NOS*	*EF1*	1.305	1.446
	*Act*	0.992	1.987
	*S7*	1.178	1.903
	*Act*/*S7*	1.081	1.945
	*EF1*/*Act*	1.138	1.695
	*EF1*/*S7*	1.240	1.659
	*EF1*/*Act*/*S7*	1.151	1.762

### *AdTEP1* and *AdNOS* are transcriptionally induced during *P. berghei* infection

**Figure 4:**
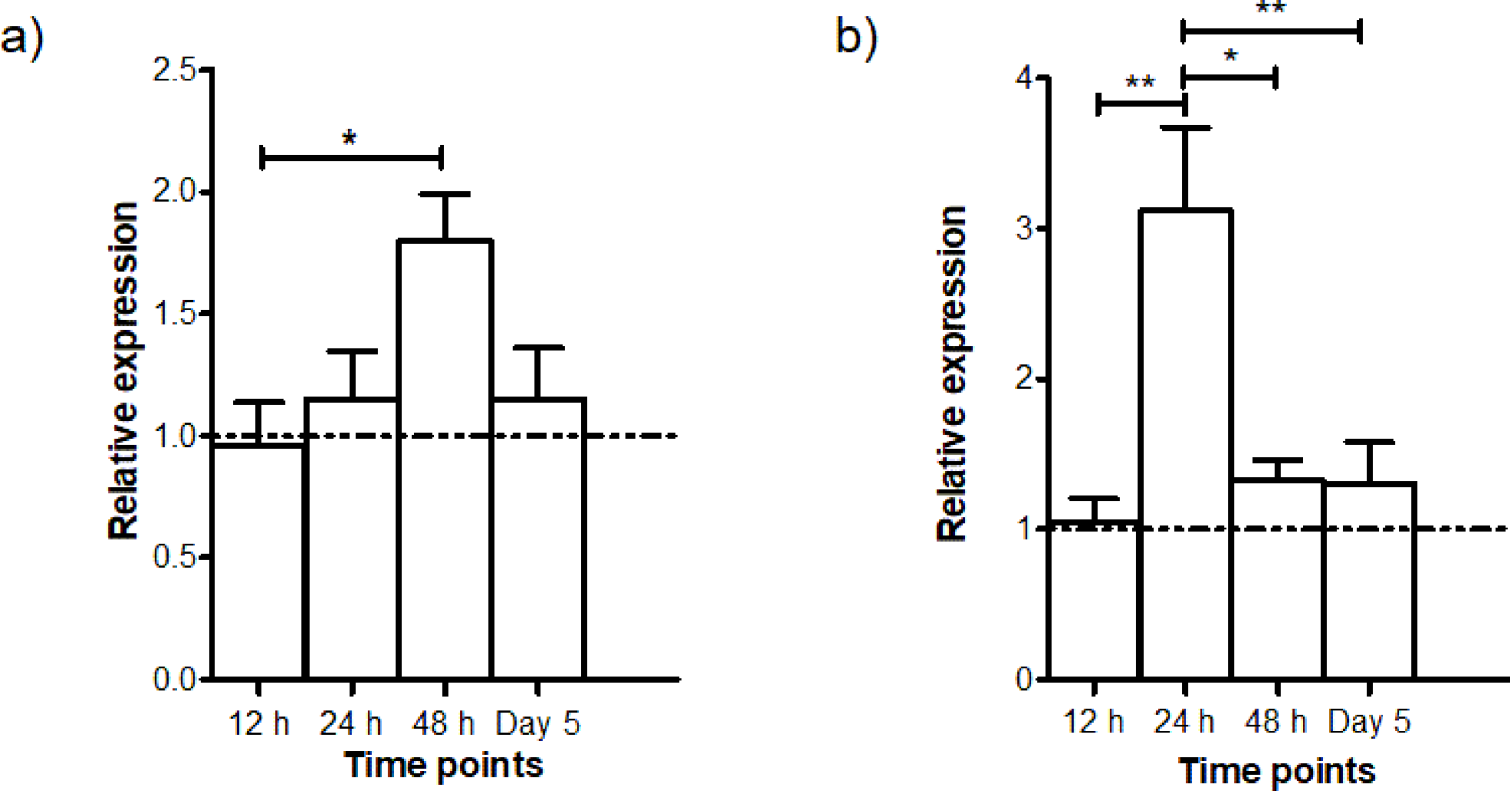
Time-point gene expression post infection (mean ± SEM) of (A) *AdNOS* and (B) *AdTEP1* in *P. berghei* ANKA-infected *An. dirus*. The dotted lines at the relative expression axes indicate the expression levels of the control group (normal blood meal). ^∗^significant (0.01 < *p* < 0.05); ^∗∗^very significant (0.001 < *p* < 0.01).

The gene expressions of *AdTEP1* and *AdNOS* at different intervals (12 h, 24 h, 48 h and Day 5) PI were studied in quadruplicates. The time points were chosen to coincide with developmental stages of the parasite: 12 h—ookinete; 24 h—early infected stage; 48 h—early oocyst; Day 5—intermediate oocyst stage (Xu et al., 2005). The control group consisted of mosquitoes which took a normal blood meal. The relative expressions of these genes are shown in Fig. 4. Transcription of *AdNOS* gene peaked to about 2-fold at 48 h PI and returned to basal level at Day 5 PI. Whereas for *AdTEP1*, there was an approximately 3-fold increase in expression 24 h PI. The upregulation of *AdNOS* and *AdTEP1* was significant but other than their respective peaks at 48 h and 24 h PI, the genes exhibited almost basal expression at all other time points.

## Discussion

*AdTEP1* and *AdNOS* proteins are homologous with those of other *Anopheles*. Structurally, *AdNOS* protein possesses the N-terminal oxygenase domain which contains a putative heme; calmodulin which links the N-terminal and C-terminal; and the C-terminal FAD/NADPH cofactor-binding domain. These have been reported to be evolutionarily conserved (Luckhart & Rosenberg, 1999). There are in fact, multiple transcripts of *NOS* in *An. stephensi* from a single copy of the gene, suggesting alternative splicing and alternative initiation events (Luckhart & Li, 2001). Investigation is underway to determine if this is also the case for *An. dirus*. On the other hand, TEP1 is structurally homologous to human complement factor C3 and contains an *α*-helical thioester region (Baxter et al., 2007). It is secreted as a full-length protein and is proteolytically cleaved to produce mature and active TEP1 (Volohonsky et al., 2017). As principal components of immunity, both *NOS* and *TEP1* genes are under selective pressure which leads to polymorphism (Le et al., 2012; Luckhart & Rosenberg, 1999). Polymorphism at the *TEP1* locus gave rise to the *Plasmodium* refractory (R) and susceptible (S) strains of *An. gambiae*. R strain is homozygous for TEP1*R1 allele, while S strains contain TEP1*S1/S2/R2 alleles (Eldering et al., 2016). The TEP1 alleles segregate in wild *An. gambiae* and *An. arabiensis* populations (White et al., 2011). It is unknown if this is also the case for *An. dirus*.

There are no universal reference genes or normalization method for qRT-PCR (Hellemans et al., 2007; Ponton et al., 2011). At least three reference genes have to be validated for their stabilities under different experimental conditions (Bustin et al., 2009; Derveaux, Vandesompele & Hellemans, 2010). *Anopheles* ribosomal protein *S7* is routinely used as a reference gene in studies similar to this. There are also gene expression studies of *Anopheles* using *actin* (Li et al., 2014; Luckhart et al., 1998) or *EF1* (Omondi, Majeed & Ignell, 2015) as a reference gene. However, most of these investigations utilized only a single reference gene for normalization, which is not recommended (Bustin et al., 2009; Vandesompele et al., 2002). Moreover, expression of *S7* of *An. stephensi* was significantly influenced by different temperature treatments (Murdock et al., 2012). Thus, total RNA concentration was included as control for background expression levels in that study. Elongation factor 1-alpha expression also fluctuates under temperature stress in the diamondback moth, *Plutella xylostella*, which is otherwise stably expressed (Fu et al., 2013). Hence, proper validation of reference genes for qRT-PCR normalization is crucial for accurate result. The three reference genes in this study were selected for validation based on previous studies. Although more candidate genes of different functional classes should be assessed (Vandesompele et al., 2002), results showed that *EF1*, *Act* and *S7* were an appropriate set of reference genes for gene expression analysis of *P. berghei*-infected *An. dirus* in this study. Since mosquitoes used in this study were of similar age, it is unlikely that the validities are confounded by ageing. Furthermore *EF1*, *Act* and *S7* have been reported to be reliable reference genes for studies on candidate age-grading genes and on different developmental stages in the parasitoid *Dastarcus helophoroides*, butterfly *Bicyclus anynana*, *Aedes aegypti* and *An. gambiae* (Dzaki et al., 2017; Pijpe et al., 2011; Wang et al., 2013a; Zhang et al., 2016).

The upregulation in *AdTEP1* expression at 24 h *P. berghei* infection corroborated well with that of another study using the *An. dirus*/*P. yoelii* model (Wang et al., 2013b). It is also similar to that of *An. gambiae* during *Plasmodium* infection. In *An. gambiae*, *TEP1* is constitutively expressed prior infection and is upregulated by 1.8–2.5-fold at 24 h post *P. berghei* infection (Blandin et al., 2004; Gupta et al., 2009). Then at 48 h PI, the *TEP1* transcript level falls back to as it was before infection (Frolet et al., 2006). The same pattern is observed in *An. dirus* (Fig. 4B). In another study, the expression depressed and peaked again at Day 4 PI. The expressions coincide with the traverse of ookinetes through the midgut cells and the development of early oocysts (Blandin et al., 2004). Since TEP1 binds to and kills *P. berghei* and *P. falciparum* midgut stages (Blandin et al., 2004), it is postulated that *TEP1* expression is induced in the fat body to replenish the protein in the hemolymph after infection (Gupta et al., 2009; Volohonsky et al., 2017).

The function and regulatory pathway of nitric oxide production in *Anopheles* are evolutionarily conserved and are shared with the vertebrates (Hillyer & Estevez-Lao, 2010). This study found that *AdNOS* expression was highest at 48 h PI while having almost basal expression at all other time points. Similarly, *An. stephensi* infected with *P. yoelli* exhibited increased mean NOS expression at 24 h PI which reached peak expression levels at 48 h PI and declined at later stages of oocyst development (Murdock et al., 2014). The repression of NOS mRNA levels through nitric oxide feedback reduces NOS protein levels to protect the host from self-induced damage (Peterson & Luckhart, 2006). Nitric oxide synthase gene expression in *An. stephensi* appears to follow the development of *P. berghei* or *P. falciparum* in the mosquito. Within 1–3 days post infection, *NOS* was transcriptionally induced coinciding with invasion and early oocyst development, while no induction was recorded on Day 6 PI when oocyst was developing. By Day 9 PI, expression was elevated and persisted till Day 14 possibly when sporozoite penetration of the salivary gland was complete (Luckhart et al., 1998). Midgut *NOS* expression was also induced in *An. culicifacies* infected with *P. vivax*, starting Day 1 until Day 15 PI (Vijay et al., 2011). In *An. aquasalis* infected with *P. vivax*, *NOS* expression was significantly induced 36 h PI, with NOS protein found mostly in midgut epithelial cells 24 h PI (Bahia et al., 2011).

The *Anopheles* immune defenses against *Plasmodium* infections have been extensively studied over the past decades, mostly using the *An. gambiae* and *An. stephensi*/*P. berghei* and *P. falciparum* infection models (Cirimotich et al., 2010; Clayton, Dong & Dimopoulos, 2014; Crompton et al., 2014). It is believed that there is a broad range of compatibility between different *Plasmodium* and mosquito strains. In addition, infection intensities and different mosquito/*Plasmodium* model influence gene expression and anti-*Plasmodium* responses, except for TEP1 activity that is not affected by infection intensity (Aguilar et al., 2005; Garver et al., 2012). Therefore, comparisons of results between studies have to be conducted with care. For example, *TEP1* silencing in *An. gambiae* (Keele strain) only doubled the median number of *P. falciparum* NF54 oocysts while *P. berghei* oocysts number increased 4–5-fold (Jaramillo-Gutierrez et al., 2009). Silencing the leucine-rich repeat protein genes of *An. gambiae* showed a profound effect on *P. berghei* infection but has no effect on the development of sympatric *P. falciparum* field isolates (Cohuet et al., 2006). Furthermore, paralog APL1A is found to be responsible for protection against *P. falciparum* while paralog APL1C is needed to protect against *P. berghei* and *P. yoelii* in *An. gambiae* (Garver, Dong & Dimopoulos, 2009; Mitri et al., 2009). Thus, distinct immune responses are elicited in response to human and rodent malaria parasite. However, the use of the less compatible *Anopheles*/*Plasmodium berghei* combination, such as the *An. dirus*/*P. berghei* model in this study, have contributed immensely to our knowledge on mosquito infection responses (Aguilar et al., 2005). Following that, more detailed studies can then be designed to investigate the efficiency and potential of these antiplasmodial responses in natural vector/parasite combinations.

Interpretation of results from this and other infection studies is further complicated by the fact that environmental temperature has significant and diverse effects on mosquito immune responses and vector competence. This temperature factor also forms complex interactions with factors such as nature of immune challenge, diet and time (Murdock et al., 2012). This makes comparison of human and rodent malaria infection difficult as their experimental temperatures (*P. berghei*: 19–21 °C; *P. falciparum*: 27 °C), optimum temperature for development and rates of development are different (Mordecai et al., 2013; Paaijmans et al., 2012). This raise the question of whether the reported disparities in immune responses are, in fact, consequences of parasitic infection, differential temperatures or both. It has been demonstrated that *An. stephensi NOS* expression is significantly affected by sampling time point and temperature. The *NOS* transcript level increased earlier at 24 h post *P. yoelii* infection when mosquitoes were maintained at warmer temperatures of 26–28 °C (Murdock et al., 2014). Besides, when *An. stephensi* was exposed to different rearing temperature, *NOS* expression was found to be induced and peaked at a later time point (24 h vs 18 h post exposure) when housed at cooler temperature (18 °C vs 22 °C) (Murdock et al., 2012). This may be a limitation of our study as the mosquitoes were placed at 20 ± 1 °C from a rearing temperature of 25–27 °C, 16–18 h prior to blood feeding. Proper studies will need to be planned to investigate and confirm this. Nonetheless, the *AdNOS* gene expression profile in the current study showed that the gene expression was basal at 12 h PI before the upregulation at 48 h PI. Additionally, complex environment drivers such as fluctuation in diurnal temperature, ambient temperature, and time of infection need to be evaluated for their effects on immunity and resistance of *An. dirus*. It has been shown that, complex interplay of these factors affects *NOS* expression in response to immune challenge (Murdock, Moller-Jacobs & Thomas, 2013).

Nevertheless, the current study demonstrates that *An. dirus* express and mount *TEP1* and *NOS* immune responses towards *P. berghei* infection, similar to the other *Anopheles*. Although the use of a rodent malaria parasite model does not necessarily reflect the temperature dependencies of the human malarias and their actual interactions with *Anopheles*, it is useful for preliminary identification and characterization of immune responses in the mosquito (Jaramillo-Gutierrez et al., 2009). Interesting findings from initial experiments using *P. berghei* in the laboratory can be subsequently validated with natural *An. dirus*/*Plasmodium* combinations. Nonetheless, our study concerning the gene expressions of TEP1 and NOS is at a preliminary stage and no protein activity was measured. More functional assays and experiments are underway to further understand the TEP1 and NOS immune responses in *An. dirus*.

## Conclusion

*AdTEP1* and *AdNOS* are homologous among the *Anopheles*. For studies comparing the gene expression of *P. berghei*-infected and normal blood fed *An. dirus*, the genes *EF1*, *Act* and *S7* are appropriate normalization controls in qRT-PCR. Using these validated reference genes, we found that *AdTEP1* and *AdNOS* expressions were highly induced respectively at 24 h and 48 h post *P. berghei* infection. Expression of *TEP1* and *NOS* in response to *Plasmodium* infection is common among the anophelines. The study employed the *An. dirus*/*P. berghei* model to characterize immune factors that could limit malaria transmission. A more comprehensive look into the *Anopheles* in different parts of the world, will enable better understanding of malaria transmission dynamics and rapid extrapolation of any novel transmission blocking strategies to allopatric *Anopheles* species.

##  Supplemental Information

10.7717/peerj.3577/supp-1 File S1 Multiple sequence alignment of (A) TEP1 and (B) NOS protein, analyzed by Clustal Omega Drosophila, *Drosophila melanogaster*; Culex, *Culex quinquefasciatus*; Aedes, *Aedes aegypti*; while the rest are all *Anopheles* species.

10.7717/peerj.3577/supp-2 File S2 Raw data for reference genes validation

10.7717/peerj.3577/supp-3 File S3 Raw data for gene expression

10.7717/peerj.3577/supp-4 File S4 DNA sequences of genes sequenced

## Additional Information and Declarations

### DNA Deposition

The following information was supplied regarding the deposition of DNA sequences: The gene sequences are uploaded as File S4. The TEP1 and NOS sequences described here have GenBank accession numbers KY465474 and KY465473, and those of EF1 and Act have the accession numbers KY022437 and KY022436.

### Data Availability

The following information was supplied regarding data availability: The raw data has been uploaded as a Supplemental File.
