# Supplementary material for: Quantitative real-time PCR analysis of Anopheles dirus TEP1 and NOS during Plasmodium berghei infection, using three reference genes
Source: PeerJ. 2017 Jul 26;5:e3577. doi: 10.7717/peerj.3577 (PMC5533154; doi:10.7717/peerj.3577)
Supplement: File S1 — Drosophila, Drosophila melanogaster; Culex, Culex quinquefasciatus; Aedes, Aedes aegypti; while the rest are all Anopheles species. [file peerj-05-3577-s001.docx]

**Supplemental file S1**

**Multiple sequence alignment of a) TEP1 and b) NOS protein**, analyzed by Clustal Omega. Drosophila: *Drosophila melanogaster*; Culex: *Culex quinquefasciatus*; Aedes: *Aedes aegypti*; while the rest are all *Anopheles* species*.*

a) TEP1 protein

Drosophila LLKINIISDTYLPYFILTVVARGNIVLSLFQEMKEKKKSQEIEFEPTFALVPQATIFVHY 60

Aedes DIVLDISCTETMTHFSYIVVTKGNIVEASNVPVAK-KKKHSLRLKMTSKMSPESRLLVYY 59

Culex EVLFEVSCTERISHFSYILVARGSIVDSGNVNVFN-KQKTSFRYRLLPQLAPKAKLIVSY 59

darlingi DISLLVTCSKGTTFYLYYVLSRGSIIDSGYVPLGKGLVSHRLQLKPSDRMLPKSTVIVAT 60

sinensis PIKYSITCTDKMQFLVYYVVSKGNIIDEGFIRPSK-TTKVQLQINANSKMIPKSKIIIAT 59

dirus MTRFLVKCSDQMGFFVYYVLSKGNIIDSGYIRPNK-QTSYLLQLNATEKMIPKSKIVVAT 59

gambiae LMRFMVTCTERMTFFVYYVMSKGNIIDAGFMRPNK-QTKYLLQLNATEKMIPKAKILIAT 59

arabiensis LMRFMVTCTERMTFFVYYVMSKGNIIDAGFMRPNK-QTKYLLQLNATEKMIPKAKILIAT 59

: . . ::::*.*: : . :. . : *:: :.:

Drosophila IID--GVLMSDEKTVDIERDFENTIEILTTN---EALPRDEVSLKVKTNPHSFVGLLGVD 115

Aedes TNR--EYLIFDDIELKFDSFNNDFKFDLND---DEYFPGQSVYIDVYASKDSYVAFSGID 114

Culex TNK--EFLIFDELDLDFDVFNNDFEFSLDHEVGNDYHPGQDIYVDLKAANDSYIAFHAID 117

darlingi IVTASQVVLYDFVELDYQALRNNFTMKLDKT---VLKPGQELRLRMTGLAGSYVALAAYD 117

sinensis LAK--DTIVYDTMDIEFKELSNNFKMTIDEQD-REVKPGRQIELIMQGRPRSYVALAAYD 116

dirus VTK--NIVVYDFVDLNFDEFRNNFELNIDED---EIKPGRQIELSMRGRPGSCVGLAAYD 114

gambiae VAG--RTVVYDYADLDFQELRNNFDLSIDEQ---EIKPGRQIELSMSGRPGAYVGLAAYD 114

arabiensis VAG--RTVVYDYADLDFQELRNNFDLSIDEQ---EIKPGRQIELSMSGRPGAYVGLAAYD 114

:: * :. . :: : * .: : : : :.: . *

Drosophila QSVLLLRSGNDLNRDLILNNLATYSTDLVILTNANINIYRSSGGCYTNPGYTNCTGSLIG 175

Aedes ESVLLVGK---ERHDFNKGDVLKELALYGATNDAEFDLFHKYG----------LFLKSTA 161

Culex QSVLHLGR---DGHVFSRDDVLEDLSQYGATEANEFDPFHTMG----------LFLRTTA 164

darlingi KSLLQFGQ----QHDLFWKDALAVLAGFHAIDDNSFDLFQRFG----------LIVQTLD 163

sinensis KSLLQHND----NHDVNWGNIEAVFDGFHDLQLNEYDKIHSMG----------LFARTLD 162

dirus ASLLAYGK----HHDLYWDDFVQVFNGFHNIDDNEFDKIHSMG----------LFARTLD 160

gambiae KALLLFNK----NHDLFWEDIGQVFDGFHAINENEFDIFHSLG----------LFARTLD 160

arabiensis KALLLFNK----NHDLFWEDIGQVFDGFHAINENEFDIFHSLG----------LFARTLD 160

::* : . : . : : *

Drosophila RTMFKNEPTKNSGPVPIVGSTRAQASLPPVRKLFPETWLFSNITDVGANGEYIIKETVPD 235

Aedes TVDTPVTRSQNARFGTLLGRT--K-QAIEIRTQFLESWLWKSFSMDGRNNFKAIEDSVPD 218

Culex QVDFPYARQQLSRFGGSVFGKRVE-KAIHIRTVFPETWLWRNYTMDGRNSKMTIASVVPD 223

darlingi GVSFDSGNTKTGREGKPTSKAKGKSTAISFRSNFLESFLWKTITM-PAAGSTELAQTVPD 222

sinensis SFKLEGASDKLGRL--ESNGSAAK--LIPYRTYFLESFLWKNLTI-PINGQRKLIENVPE 217

dirus DIIFDGANDKSARAGSQSNKAPTK--LVEFRTNFAESFLWKNVTI-GRQGTRSLIEVVPD 217

gambiae DILFDSANEKTGRNALQSGKPIGK--LVSYRTNFQESWLWKNVSI-GRSGSRKLIEVVPD 217

arabiensis DILFDSANEKTGRNALQSGKPIGK--LVSYRTNFQESWLWKNVSI-GRSGSRKLIEVVPD 217

: . : *. * *::*: . : . : . **:

Drosophila TLTSWVITGFSLSPQSGLAVTRNPSRIRVFQPFFITTNLPYSVKRGEVIAIPVIVFNYLG 295

Aedes TITTYHVSGFALSPTLGLGVIQQPVSFTVRKKFYLVANLPYSIKRGEVALIQVTVFNFLG 278

Culex TVTSWTVTGFALSPTYGLGIMQETREFTVNQPFYIIANLPYSIKRDEVAVIHVTVFNFLG 283

darlingi TTTAWQLTGFSIDPVYGLGIIEQPLQFITVQQFYIVDSLPYSIKRGEAVVLQFSLFSQLS 282

sinensis STTAWSLTGFSIDPVYGLGIIKEPIEFTTVQSFYIVDNLPYSIKRGEAVALQFTLFNNLG 277

dirus TTTSWYLKGFSIDPTYGLGIIKKPIVFKTVQPFYIVENLPYSIKRGEAVVLQFTLFNSLG 277

gambiae TTTSWYLTGFSIDPVYGLGIIKKPIQFTTVQPFYIVENLPYSIKRGEAVVLQFTLFNNLG 277

arabiensis TTTSWYLTGFSIDPVYGLGIIKKPIQFTTVQPFYIVENLPYSIKRGEAVVLQFTLFNNLG 277

: *:: :.**::.* **.: .: : . : *:: .****:**.*. : . :*. *.

Drosophila MDVKAKVLMDNSDGQYEFIETTNKNVSQYLRGVRRKKTLWIPANTGRGISFMIRPKKVGL 355

Aedes SSVTTDVTLFNKRDEIEFVEKASTNNTH------RTKAVIAPNNNGKPVSFMVKAKKLGQ 332

Culex NTLTTDVTLFNKNDEIEFVEKSSDDPTR------RTKAIIVPGNNGKPISFLIKAKKLGE 337

darlingi TVQTAQVTLYTGDNRTEIVGQPVTAQS-------LTKTVTVPANVGVPVTFLVKARAIGE 335

sinensis GEYIADVTMYNVGNQIEFVGQPAGTSS-------YTKSISVKPKVGVPISFLVKAKKLGE 330

dirus AEYIADVTLLNVANQTEFVGRPLEDVS-------YTKSVSVLPNVGVPVSFLVKARKLGE 330

gambiae AEYIADVTLYNVANQTEFVGRPDTDLS-------YTKSVSVPPKVGVPISFLIKARKLGE 330

arabiensis AEYIADVTLYNVANQTEFVGRPDTDLS-------YTKSVSVPPKVGVPISFLIKARKLGE 330

:.* : . .. *:: : .*:: : * ::*::: : :*

Drosophila TTLKITAIS--KYAGDRLHQILKVEADGVQKYVNKAVLINVQRLNRRSLAPPEKTIIIEK 413

Aedes IAIKFQAEN--LLETDALEHMLRVTPESHRYEKNVARFIQLPTHSKVPFD-----VKLDI 385

Culex IAIKIQAVN--PLKTDSVEHMLRVIPESHMHEVNEARFIDLPKNTVQNFQ-----IQVNI 390

darlingi MVVRIKAVCSSSEVQDELEKVIRVVPESLIQRRHVSRFFSHSNYANQTFA-----VVLDI 390

sinensis IAVRLKASIMLGQETDAIEKVIRVLPESLVEKRMESRIFSQNKYVNTSFP-----IILDI 385

dirus MVVRVKAWIMNGLETDGLEKVIRVLPESLVQPRMLSQFFCFDEYKNQTFQ-----FNLDI 385

gambiae MAVRVKASIMLGHETDALEKVIRVMPESLAQPKMDTSFFCFDDYKNQTFP-----FNLDI 385

arabiensis MAVRVKASIMLGHETDALEKVIRVMPESLAQPKMDTSFFCFDDYKNQTFP-----FNLDI 385

.::. * * :.::::* :. : :: : . ::

Drosophila ADNVIEGSETVEFEVCGTSQAPQLEHLDDLVHLPCGCGEQNMFNFVPSILALSYLKAKNR 473

Aedes PKNIDEGSAQIKFTLDPDILGTTISNLDGLIRKPSGCGEQNMLHFVPNIVVLDYLNETNT 445

Culex PREIDEGSARIKFTLDPDILGTAIKNLESLIRLPTGCGEQNMMRFVPNIVVLDYLSETGT 450

darlingi DKQADERSRRIEFTLVPNLLTSVLKNLGNLLSVPSGCGEQNMVRYVPNILVLDYLTAIGS 450

sinensis NKNADVGSAQISFYVNPNILTTVVQNLGELLDVPSGCGEQNMVNFVPNIIVLDYLTAIGS 445

dirus NKLADKGSEKIKFRVNPNLLSSVIDNLDHLLAVPTGCGEQHMVKFVPNIVALDYLTAVGS 445

gambiae NKKADNGSKKIEFRLNPNLLTMVIKNLDNLLAVPTGCGEQNMVKFVPNILVLDYLYATGS 445

arabiensis NKKADNGSKKIEFRLNPNLLTMVIKNLDNLLAVPTGCGEQNMVKFVPNILVLDYLYATGS 445

* :.* : :.:* *: * *****:*..:**.*:.*.** .

Drosophila QDQEIENKAKRYVETGYQIELNYKRNDGSFSAWGQHDALGSTWLTAYVIRSFHQAAKYI- 532

Aedes AAEDVRTKAINFLSSGYQNQLRYKRSDGAFSVWGQSHA-GSTFLTAFVAKSFKIAAKYI- 503

Culex IRDDIKEKAIGYLKSGYQNQMKYKLSDGSFGIWASSR--GGTFLTAFVAKSLKIADKYM- 507

darlingi GETHLISRATELLRVGHQNQLRFRQPDGSFGLWSASG--GGVFLTAFVGSSMKIASKYIA 508

sinensis KETSVINKATGMLRTGYQNQMRYRQTDGSFGVWSSGG--GSVFLTAFVAKSMQIASKYIS 503

dirus KQKTIIDKATNMLRQGYQNQMRYRQADGSFGVWGHSG--GSVFLTAFVAKSMQTASKYI- 502

gambiae KEQHLIDKATNLLRQGYQNQMRYRQTDGSFGVWEKSG--SSVFLTAFVATSMQTASKYMN 503

arabiensis KEQHLIDKATNLLRQGYQNQMRYRQTDGSFGVWEKSG--SSVFLTAFVATSMQTASKYMN 503

: :* : *:* ::.:: **:*. * ...:***:* *:: * **:

Drosophila DIDKNVLVAGLDFLVSRQSTDGKFKELGMVIHNSHGS-----PLALTSFVLLTFFENEEY 587

Aedes QVDKSIVDAAFDWLAKQQQSDGRFPEVGQVIHADMQGGLRNNGFALTAYVLIAFAENEEV 563

Culex SVDIDVVNKAFAWLAGKQQPDGRFTEVGDVAHADMQGGLRSTSYALTAYVLAAFLETKSI 567

darlingi DVEPSIVRQAFDWLATKQHTSGRFDAAGAMYHRDMQGGLRD-GIALTAYVLIAFLENPEA 567

sinensis EVDGTMVANAFQWLATKQKYDGRFDEVGSVIHKDMQGGLRN-GIALTSYVLIAFLENENA 562

dirus DVDKALVERAYEWLASKQSFSGRFDEVGSVIHQDMQGGLRN-GVALTSYVLTALLEHQGS 561

gambiae DIDAAMVEKALDWLASKQHSSGRFDETGKVWHKDMQGGLRN-GVALTSYVLTALLENDIA 562

arabiensis DIDAAMVEKALDWLASKQHSSGRFDETGKVWHKDMQGGLRN-GVALTSYVLTALLENDIA 562

.:: :: . :*. :* .*:* * : * . . ***::** :: *

Drosophila MPKYKHVIDRAVEFVVTEVHQSNEPYDLAIAALALSLAR--NRNAYKVLDKLDKLATRRG 645

Aedes YRKYRSQLIKTTSYIANNLDNMENPYDLSLSTYALMLAN--HGKRTEFLDKLVEISIFDS 621

Culex AQTHSDVIRRSANYLMSNFDNMNNVYDLALTTYALSLQS--PASSQKFFDKLVESSTYDS 625

darlingi ARNHPKVIEKGMQYVTEAVPRVTDAYDLSIATYALWLAS--HPLRFETLDRLTGLSTTKS 625

sinensis KVTHAKVIENSITYIARHLPQVTDIYDLSIATYAMMLSNYNQNQKENFMKKLIDRSTVYG 622

dirus RTTYAQHIQKALHYLNNQLASINEPYDLSIATYALMLGK--HINKNEALKKLVGLSTPLN 619

gambiae KVKHAVVIQNGMNYLSNQLAFINNPYDLSIATYAMMLNG--HTMKKEALDKLIDMSISDN 620

arabiensis KVKHVVVIQNGMNYLSNQLALINNPYDLSIATYAMMLNG--HTMKKEALDKLIDMSISDN 620

.: : . :: . : ***:::: *: * : :.:* : .

Drosophila DHKWWTGSDKCKSSEVETTSYVLLALLEHNISDEPKPIVDWLISKRNSNGGFVSSQDTVV 705

Aedes NQTERYWDS--KPVDIEVAGYALLSYVAAGDLLHATPIMRWLNKQRYGLGGFPGTQDTFV 679

Culex TNKVRSWSY--KSLGVEIAGYALLSYVQREQILDATPIMRWLTSQRYDLGGFPGTQDTYV 683

darlingi NGTERYWAR--AANGIETTAYALLSYVLAERYLDGVPIMRWLVGQRYETGSFPRTQDTFV 683

sinensis NGTIRYWAR--HSHEIETTGYALLAMVQDEKYPDSIPVMRWLVNQRHPKGSFPRTQDTFV 680

dirus NGTERYWNT---ANKIEATAYALLSFVAAEKYIEGIPIMRWLVDQRFVTGSFPRTQDTFV 676

gambiae NKKERYWGT---TNQIETTAYALLSFVMAEKYLDGIPVMNWLVNQRYVTGSFPRTQDTFV 677

arabiensis NKKERYWGT---TNQIETTAYALLSFVMAEKYLDGIPVMNWLVNQRYVTGSFPRTQDTFV 677

. :* :.*.**: : . *:: ** :* *.* :*** *

Drosophila GIMALTKYELQSHASTEAIDIEFWHLNEDKKHVRVTKENEFKVQTHQLPENTNEVKLLAK 765

Aedes GLKALATFAAKVSSGRNDYRVTIIHEPNRRRTFDVDRHNAFNIQELDIPNNIRKMRVEVV 739

Culex GLKALAKFAAKASAHRNDYRVTVRPKKEKLLTFDVDKK-VLSIQELDLDSSSRSIDVEVS 742

darlingi GLKALCKMAEKVAPARNDYSIQLVYPQRR-REFRVSSGDLEQMSYDEFTDATNKLEFHVQ 742

sinensis GLKSLTKLAEVISPSRNDYSIQLKYKTQS-KMFFITSTEVEQRKFEDIPSEVRKLDMIVA 739

dirus GLKALTELAEKISPSRNDYSIQVTTKKLP-KRYFINSKDVVPLEEEKFPXDTRKMEVNVG 735

gambiae GLKALTKLAEKISPSRNDYTVQLKYKKNT-KYFNINSEQIDVQNFLEIPEDTKKLEINVG 736

arabiensis GLKALTKLAEKISPSRNDYTVQLKYKKNT-KYFNINSEQIDVQNFLEIPEDTKKLEINVG 736

*: :* : : . : . .: ..: . .

Drosophila GQGRAQVQLTYRYNVATKEARPSFKLTTTVKKSHK--GRLILGICGTYTPIAASERNKTT 823

Aedes GIGNGYFQVAYQYYQNIQVAKPSFSLTIDQLNTTT-EHMQQLDVCVKYIPKEAYQK---S 795

Culex GIGKGVFQVAYQYYQNILKEKSSFDLSVNLMNTTT-YYRQQLHVCVKFNPKEAYQY---S 798

darlingi GTGFGLLQVSYQYAMELSKFANQFVLDVTKRDSGSTETKLVLDVCTSYKPKLTNER---S 799

sinensis GMGFGLLDIKYEYTMDLRNYKNRFDLTLEKLNNNS-DYELKLRICANFIPKLVNER---S 795

dirus GLGFGLLEVIXEYTLNLQNFSHRFDLNLEKQDTGS-DYALQLKVCTSYIPKLSNER---S 791

gambiae GIGFGLLEVIYQFDLNLVNFEHRFKLDLEKQNTGS-DYELRLRVCANYIPELTDSQ---S 792

arabiensis GIGFGLLEVIYQFDLNLVNFEHRFKLDLEKQNTGS-DYELRLRVCANYIPELTDSQ---S 792

* * . .:: .: * * .. . * :* .: * . :

Drosophila NMALMQVQLPSGYVCDIEPFADIEAISDVKRVETKNEDTEVHIYFEKLSPGDRKCLTLEA 883

Aedes NMALVEIFLPSGLVADSDAITDKT--GGIRRIERRFSDTSVVIYYDNLGP-EDECFRVTA 852

Culex NMVLVEVFFPSGIVADDDAVQDLSSNRAIQKTELRFGGTSLVVYYTRLGV-QKNCFTVTA 857

darlingi NMVLVEVNFPSGYVAEKSPLSATTTVNPIRNVEIRFGGTSVVLYYDNMGT-ERNCFAVTA 858

sinensis NMALVEVNFPSGYVVDPRPITEATTVNPIRNTEIRFGGTAVVAYYNNMGT-EKNCFVITA 854

dirus NMALVEVXLPSGYVADRNPISQQXTINPIQNIELRYGSTSIVAYYNNMGT-EKNCFTVTA 850

gambiae NMALIEVTLPSGYVVDRNPISEQTTVNPIQNMEIRYGGTSVVLYYYKMGT-ERNCFTVTA 851

arabiensis NMALIEVTLPSGYVVDRNPISEQTTVNPIQNMEIRYGGTSVVLYYYNMGT-ERNCFTVTA 851

**.*::: :*** * : . ::. * : .* : *: .:. : :*: : *

Drosophila IYTHAVANLKPSWVRLYDYYA---TERSATEFYHVDT-SLCDICHGNECGNMC------- 932

Aedes YRRYKIALHLPSYIIVYDYYN---SERFAIQQYEGKVLQLCDICEDEDCETLSCENSSK- 908

Culex ERRFNVALHRPTYVVVYDYYDNKQSDRFAIQSYEGKVMQLCDVCEDEDCVTLACPPSTAS 917

darlingi YRRTRVALNRPAYVIVHDYYE---PEKNAVKTYEVERRDVCELCDGNNECREVCQGG--- 912

sinensis YRRFKVALKRPAYVLVHDYYE---PKLNAIQVYDVDQENLCDICDK-EDCPPECNK---- 906

dirus YKRFKVALIRPAYVVVYDYYN---ADLNAIKLYEVFSQKICDLCEE-GSCPDECSFSPIA 906

gambiae YRRFKVALKRPAYVVVYDYYN---TNLNAIKVYEVDKQNVCEICEE-EDCPAECKK---- 903

arabiensis YRRFKVALKRPAYVVVYDYYN---TNLNAIKVYEVDKQNVCEICEE-EDCPAECKK---- 903

:* *::: ::*** . * : *. .:*::*.

Drosophila ---- 932

Aedes ---- 908

Culex G--- 918

darlingi ---- 912

sinensis ---- 906

dirus AETT 910

gambiae ---- 903

arabiensis ---- 903

b) NOS protein

Drosophila ------------------------------------------------------------ 0

Culex ------------------------------------------------------------ 0

darlingi ------------------------------------------------------------ 0

aquasalis ------------------------------------------------------------ 0

Aedes ------------------------------------------------------------ 0

pseudopunctipennis ------------------------------------------------------------ 0

sinensis MSQHLSSMLEHLKMGSLMSKGGGGAGGAKQQQVQPVSAGSTSSPNVAVVGNGSIAGAPNG 60

gambiae ------------------------------------------------------------ 0

dirus ------------------------------------------------------------ 0

stephensi ------------------------------------------------------------ 0

Drosophila ------------------------------------------------------------ 0

Culex ------------------------------------------------------------ 0

darlingi ----------------------------------------------MRHRALNLGKFPAT 14

aquasalis ------------------------------------------------------------ 0

Aedes ------------------------------------------------------------ 0

pseudopunctipennis ------------------------------------------------------------ 0

sinensis CANNNVHCSSGAPLSNHQNNNLHHHNHNNNCQNGGAGMTGAKIGADTPVIVERGRCTTTV 120

gambiae ------------------------------------------------------------ 0

dirus ------------------------------------------------------------ 0

stephensi -------------------------------------------MADTTTVVV--ERREVA 15

Drosophila ------------------------------------------------------------ 0

Culex ------------------------------------------------------------ 0

darlingi EHHNRRNGRYGNRDEGGWSEQVRQPLTGVGEGFSVLSSTSDERYGISYLESSFSLKLLGQ 74

aquasalis ------------------------------------------------------------ 0

Aedes ------------------------------------------------------------ 0

pseudopunctipennis ------------------------------------------------------------ 0

sinensis VEVSGKSANHVGEDRRGYDVSRKRCSISVQQHGVAGGGGSEAGGNSRSNYRELSPASLRI 180

gambiae ------------------------------------------------------------ 0

dirus ------------------------------------------------------------ 0

stephensi EGRESSKANHIGEERRGYDVSRKRCSISVHGGGTEGG-----GGNMRTNYRELSPASLRI 70

Drosophila ------------------------------------------------------------ 0

Culex ------------------------------------------------------------ 0

darlingi WFNLQPSEAIDARWRSTEEKDVIDCTAGSSFGQQSWTITSYAASMNCRRFGHQNVVPYVA 134

aquasalis ------------------------------------------------------------ 0

Aedes ------------------------------------------------------------ 0

pseudopunctipennis ------------------------------------------------------------ 0

sinensis HRKSSHDIRNTLLGPDGEALHLHDPSGKGGDGLGKMPTVVKPIKLKSIITKAESYDTMHG 240

gambiae ------------------------------------------------------------ 0

dirus ------------------------------------------------------------ 0

stephensi HRKSSHDIRNTLLGPDGEVLHLHDPSGKGGDGMGKMPAVVKPIKLKSIVTKAESYDTMHG 130

Drosophila -----------------MNIGNAAVEARKSDLILEHAKDFLEQYFTSIKRT--------- 34

Culex ------------------------------------------------------------ 0

darlingi RTSQVMLCSREVCLGSVLTLHNAGTEPRKSDVVLQHAKDFLDQYYSSIRRFTGYSFYGEA 194

aquasalis ------------------------------------------------------------ 0

Aedes -----MHCSREVCMGSVMLPNIVGTEPRKPDIVLQHAKEFLDQYYSSIRRL--------- 46

pseudopunctipennis ------------------------------------------------------------ 0

sinensis KASDVMSCSREVCMGSVMTPHLIGTESRKPDVVQQHAKDFLDQYYSSIRRL--------- 291

gambiae -----------VCMGSVMTPHLVGTEARKSEIVQQHAKDFLDQYYSSIRRL--------- 40

dirus ------------------------------------------------------------ 0

stephensi KASDVMSCSREVCMGSVMTPHVIGTETRKPEIVQQHAKDFLDQYYSSIRRL--------- 181

Drosophila -------------------------------------------------------SSTAH 39

Culex ------------------------------------------------------------ 0

darlingi ISLEQYFPGENTEYSEGREYRAKDAKLFFNESHKIKQQSSSSSSSSSSPSPASPLKSPAH 254

aquasalis ------------------------------------------------------------ 0

Aedes -------------------------------------------------------KSPAH 51

pseudopunctipennis ------------------------------------------------------------ 0

sinensis -------------------------------------------------------KSPAH 296

gambiae -------------------------------------------------------KSPAH 45

dirus ------------------------------------------------------------ 0

stephensi -------------------------------------------------------KSPAH 186

Drosophila ETRWKQVRQSIETTGHYQLTETELIYGAKLAWRNSSRCIGRIQWSKLQ------------ 87

Culex ------------------------------------------------------------ 0

darlingi DTRWQQIQKEVAATGAYHLTETELIYGAKLAWRNSARCIGRIQWSKLQQIRSQLHLAERL 314

aquasalis ------------------------------------------------------------ 0

Aedes ESRWQQVQKEVDSTGSYQLTETELIYGAKLAWRNSSRCIGRIQWSKLQ------------ 99

pseudopunctipennis ------------------------------------------------------------ 0

sinensis ESRWQQVQKEVEATGSYHLTETELIYGAKLAWRNSSRCIGRIQWSKLQ------------ 344

gambiae DTRWQQVQKEVEATGSYHLTETELIYGAKLAWRNSSRCIGRIQWSKLQ------------ 93

dirus ------------------------------------------------------------ 0

stephensi DSRWQQVQKEVEATGSYHLTETELIYGAKLAWRNSSRCIGRIQWSKLQ------------ 234

Drosophila -------------VFDCRYVTTTSGMFEAICNHIKYATNKGNLRSAITIFPQRTDAKHDY 134

Culex ------------------------------------------------------------ 0

darlingi IGGSTDTNDQTTNVFDCRYVTTTSGMFEAICNHIKYATNKGNLRSAITIFPQRTDGKHDY 374

aquasalis ------------------------------------------------------------ 0

Aedes -------------VFDCRYVTTTSGMFEAICNHIKYATNKGNLRSAITIFPQRTDGTHDY 146

pseudopunctipennis ------------------------------------------------------------ 0

sinensis -------------VFDCRYVTTTSGMFEAICNHIKYATNKGNLRSAITIFPQRTDGKHDY 391

gambiae -------------VFDCRYVTTTSGMFEAICNHIKYATNKGNLRSAITIFPQRTDGKHDY 140

dirus -------------------------MFEAICNXIKYATNKGNLRSAITIFPQRTDGKHDY 35

stephensi -------------VFDCRYVTTTSGMFEAICNHIKYATNKGNLRSAITIFPQRTDGKHDY 281

Drosophila RIWNNQLISYAGYKQADGKIIGDPMNVEFTEVCTKLGWKSKGSEWDILPLVVSANGHDPD 194

Culex ------------------------------------------------------------ 0

darlingi RIWNQQIISYAGYKNADGKIIGDPANVEFTDFCIKLGWKSKRTEWDILPLVVSANGHDPD 434

aquasalis ------------------------------------------------------------ 0

Aedes RIWNAQLISYAGYKGQDGKIVGDPMNVEFTDFCIKLGWKSKGTEWDILPVVVSANGHDPD 206

pseudopunctipennis ------------------------------------------------------------ 0

sinensis RIWNNQIISYAGYKSADGKIIGDPANVEFTDFCVKLGWKSKRTEWDILPLVVSANGHDPD 451

gambiae RIWNNQIISYAGYKNADGKIIGDPANVEFTDFCTKLGWKSKRTEWDILPLVVSANGHDPD 200

dirus RIWNNQIISYAGYKNADGKIIGDPANVEFTDFCVKLGWKSKRTEWDILPLVVSANGHDPD 95

stephensi RIWNNQIISYAGYKNADGKIIGDPANVEFTDFCVKLGWKSKRTEWDILPLVVSANGHDPD 341

Drosophila YFDYPPELILEVPLTHPKFEWFSDLGLRWYALPAVSSMLFDVGGIQFTATTFSGWYMSTE 254

Culex ------------------------------------------------------------ 0

darlingi YFDYPPDLILQVPLSHPQFKWFAELDLRWYAVPMVSGMLFDCGGIQFTATAFSGWYMSTE 494

aquasalis ------------------------------------------------------------ 0

Aedes YFDYPSELILEVPFSHPQYKWFAEMGLRWYALPAVSGMLFDCGGIQFTATSFSGWYMSTE 266

pseudopunctipennis ------------------------------------------------------------ 0

sinensis YFDYPPELILEVPLSHPQFKWFAELNLRWYAVPMVSSMLFDCGGIQFTATAFSGWYMSTE 511

gambiae YFDYPPELILEVPLSHPQFKWFAELNLRWYAVPMVSSMLFDCGGIQFTATAFSGWYMSTE 260

dirus YFDYPPELILEVPLSHPQFKWFAELNLRWYAVPMVSSMLFDCGGIQFTATAFSGWYMSTE 155

stephensi YFDYPPELILEVPLSHPQFKWFAELNLRWYAVPMVSSMLFDCGGIQFTATAFSGWYMSTE 401

Drosophila IGSRNLCDTNRRNMLETVALKMQLDTRTPTSLWKDKAVVEMNIAVLHSYQSRNVTIVDHH 314

Culex ------------------------------------------------------------ 0

darlingi IGCRNLCDTNRRNLLEPIAIKMGLDTRNPTSLWKDKALVEINIAVLHSYQSRNITIVDHH 554

aquasalis ------------------------------------------------------------ 0

Aedes IGCRNLCDINRRNLLEPIAVKMGLDTRNPTSLWKDKTLVEINIAVLHSFQSRNITIVDHH 326

pseudopunctipennis ------------------------------------------------------------ 0

sinensis IGCRNLCDANRRNLLEPIAIKMGLDTRNPTSLWKDKALVEINIAVLHSYQSRNITIVDHH 571

gambiae IGCRNLCDANRRNLLEPIAIKMGLDTRNPTSLWKDKALVEINIAVLHSFQSRNITIVDHH 320

dirus IGCRNLCDTNRRNLLEPIAIKMGLDTRNPTSLWKDKALVEINIAVLHSYQSRNITIVDHH 215

stephensi IGCRNLCDANRRNLLEPIAIKMGLDTRNPTSLWKDKALVEINIAVLHSYQSRNITIVDHH 461

Drosophila TASESFMKHFENESKLRNGCPADWIWIVPPLSGSITPVFHQEMALYYLKPSFEYQDPAWR 374

Culex ------------------------------------------------------------ 0

darlingi TASESFMKHCENETKLRNGCPADWIWIVPPMSASVTPVFHQEMALYYLRPSFEYQESAMK 614

aquasalis ------------------------------------------------------------ 0

Aedes TASESFMKHYENETKLRNGCPADWVWIVPPMSASATPVFHQEMALYYLRPSFEYQESALK 386

pseudopunctipennis ------------------------------------------------------------ 0

sinensis TASESFMKHFENETKLRNGCPADWIWIVPPMSASVTPVFHQEMAVYYLRPSFEYQESAMK 631

gambiae TASESFMKHYENETKLRNGCPADWVWIVPPMSASITPVFHQEMALYYLRPSFEYQESAMK 380

dirus TASESFMKHFENETKLRNGCPADWIWIVPPMSASVTPVFHQEXXVYYLRXSFEYXETAXK 275

stephensi TASESFMKHFENETKLRNGCPADWIWIVPPMSASVTPVFHQEMAVYYLRPSFEYQESAMK 521

Drosophila THVWKKGRGES-KGKKPRRKFNFKQIAR-------------------------------- 401

Culex ------------------------------------------------------------ 0

darlingi THCWKKEGGRQQGNKKPRRKFNFKQIASVQTIQTLTRRPRPVDKTDNRVIRYNEGKQLDL 674

aquasalis ------------------------------------------------------------ 0

Aedes THIWKKGRDSS-KNKKPRRKFNFKQIAR-------------------------------- 413

pseudopunctipennis ------------------------------------------------------------ 0

sinensis THIWKKGRDSA-KNKKPRRKFNFKQIAR-------------------------------- 658

gambiae THIWKKGRDSA-KNKKPRRKFNFKQIAR-------------------------------- 407

dirus THIWKKGRDSA-KXKKPRRKFNFKQIAR-------------------------------- 302

stephensi THIWKKGRDSA-KNKKPRRKFNFKQIAR-------------------------------- 548

Drosophila ------------------------------------------------------------ 401

Culex ------------------------------------------------------------ 0

darlingi PSKVGHIISISDDDIPASGNHAQLTAAATPNAVHHMLNVLRLTPFTAAHLQLQLKMLLVD 734

aquasalis ------------------------------------------------------------ 0

Aedes ------------------------------------------------------------ 413

pseudopunctipennis ------------------------------------------------------------ 0

sinensis ------------------------------------------------------------ 658

gambiae ------------------------------------------------------------ 407

dirus ------------------------------------------------------------ 302

stephensi ------------------------------------------------------------ 548

Drosophila -----------------------AVKFTSKLFGRALSKRIKATVLYATETGKSEQYAKQL 438

Culex ------------------------------------------------------------ 0

darlingi EIAKLSGSGKSTRRSLAFSFGGTAVKFTSKLFGRALSRRIKATVLYATETGRSEAYARQL 794

aquasalis ------------------------------------------------------------ 0

Aedes -----------------------AVKFTSKLFGRALSRRIKATVLYATETGRSEQYAKQL 450

pseudopunctipennis ------------------------------------------------------------ 0

sinensis -----------------------AVKFTSKLFGRALSRRIKATVLYATETGRSEQYARQL 695

gambiae -----------------------AVKFTSKLFGRALSRRIKATVLYATETGRSEQYARQL 444

dirus -----------------------AVKFTSKLFGRALSRRIKATVLYXTETGRSEQYARQL 339

stephensi -----------------------AVKFTSKLFGRALSRRIKATVLYATETGRSEQYARQL 585

Drosophila CELLGHAFNA--------------------------QIYCMSDYDISSIEHEALLIVVAS 472

Culex ------------------------------------------------------------ 0

darlingi VELLGHAFNAQALAFVPGLACENPIEYELRPYRRVREIYSMADYDISSIEHEALLLLVAS 854

aquasalis ------------------------------------------------------------ 0

Aedes VELLGHAFNA--------------------------QIYCMSDYDISSIEHEALLLVVAS 484

pseudopunctipennis ------------------------------------------------------------ 0

sinensis VELLGHAFNA--------------------------QIYCMSDYDISSIEHEALLLVVAS 729

gambiae VELLGHAFNA--------------------------QIYCMSDYDISSIEHEALLLVVAS 478

dirus VELLGHAFNA--------------------------QIYCMSDYDISSIEHEALLLVVAS 373

stephensi VELLGHAFNA--------------------------QIYCMSDYDISSIEHEALLLVVAS 619

Drosophila TFGNGDPPENGELFSQELYAMRVQESSEHG--LQDSSIGSSKSFMKASSRQEFMKLPLQQ 530

Culex -MDLQNSNLMLELFAQDLYAMRLHETGHSQVHSELSIAASSKSFIKANSRSDMNRH---- 55

darlingi TFGNGDPPENGQLFAQDLYAMKLHESGNNHGHSELSIAASSRSFIKANSRSELPKCGPMG 914

aquasalis ------------------------------------------------------------ 0

Aedes TFGNGDPPENGELFAQDLYAMKLHEGGHNQAHSELSIAASSKSFIKANSRSDMGKLGQ-N 543

pseudopunctipennis --AKGDPPENGELFAQDLYAMKLHESGHHQAHSELTIAASSKSFIKANSRSDLGKFGHLG 58

sinensis TFGNGDPPENGELFAQDLYAMKLHESGHHQAHSELTIAASSKSFIKANSRSDLGKFGHLG 789

gambiae TFGNGDPPENGELFAQDLYAMKLHESGHHQAHSELTIAASSKSFIKANSRSDLGKFGPMG 538

dirus TFGNGDPPENGELFAQDLYAMKLHESGHHQAHSELTIAASSKSFIKTNSRSDLGKFGPAG 433

stephensi TFGNGDPPENGELFAQDLYAMKLHESGHHQAHSELTIAASSKSFIKANSRSDLGKFGPMG 679

Drosophila VKRIDRWDSLRGSTSDTFTEETFGPLSNVRFAVFALGSSAYPNFCAFGQYVDNILGELGG 590

Culex -RKIDRLDSLRGSTTDTLSEETFGPLSNVRFAVFALGSSAYPNFCAYGKYIDNILDELGG 114

darlingi GRKMDRLDSLRGSTTDTLSEETFGPLSNVRFAVFALGSSAYPNFAAFGKYLDNILGELGG 974

aquasalis ------------------------------------------------------------ 0

Aedes NRKIDRLDSLRGSTTDTLSEETFGPLSNVRFAVFALGSSAYPNFCAYGKYIDNILGELGG 603

pseudopunctipennis GRKIDRLDSLRGSTTDTLSEETFGPLSNVRFAVFALGSSAYPNFCAFGKYLDNILGELGG 118

sinensis GRKIDRLDSLRGSTTDTLSEETFGPLSNVRFAVFALGSSAYPNFCAFGKYIDNILGELGG 849

gambiae GRKIDRLDSLRGSTTDTLSEETFGPLSNVRFAVFALGSSAYPNFCAFGKYIDNILGELGG 598

dirus ARKIDRLDSLRGSTTDTLSEETFGPLSNVRFAVFALGSSAYPNFCAFGKYIDNILGELGG 493

stephensi GRKIDRLDSLRGSTTDTLSEETFGPLSNVRFAVFALGSSAYPNFCAFGKYIDNILGELGG 739

Drosophila ERLLRVAYGDEMCGQEQSFRKWAPEVFKLACETFCLDPEESLSDASLALQNDSLTVNTVR 650

Culex ERLMRMATGDEICGQEQAFRKWAPEVFKIACETFCLDPEETLSDAAFSMQT-ELTENTVR 173

darlingi ERILKLATGDEICGQEQAFRKWAPEVFKVACETFCLDPEETLSEGVFTMQN-ELTEQTVR 1033

aquasalis ------------------------------------------------------------ 0

Aedes ERLMKMATGDEICGQEQAFRKWAPEVFKIACETFCLDPEETLSDAAFALQS-ELSENTVR 662

pseudopunctipennis ERMMKLATGDEICGQEQAFRKWAPEVFKTACETFCLDPEETLSDMSFGLQS-ELSENTVR 177

sinensis ERMMKMATGDEICGQEQAFRKWAPEVFKIACETFCLDPEETLSDAAFALQS-ELSENTVR 908

gambiae ERLMKMATGDEICGQEQAFRKWAPEVFKIACETFCLDPEETLSDAAFALQS-ELSENTVR 657

dirus ERLMKMATGDEICGQEQAFRKWAPEVFKIACETFCLDPEETLSDAAFALQS-ELSENTVR 552

stephensi ERLMKMATGDEICGQEQAFRKWAPEVFKIACETFCLDPEETLSDAAFALQS-ELSENTVR 798

Drosophila LVPSANKGSLDSSLSKYHNKKVHCCKAKAKPHNLTRL-SEGAKTTMLLEICAPGLEYEPG 709

Culex FAPVTEYESLDKALSKYHNKKATECTLKRNAINLHEGTNGSERSTILVEIIAEGMAYEPG 233

darlingi YTPVSEEEPLDRALSKYHNKKAIECRVKRTPISLQDS--KTEKSTISVEIVAEGVDYEPG 1091

aquasalis ------------------------------------------------------------ 0

Aedes FAPVNEYERLDVALSKFHNKKATECTLKRKALNLHEGTNGSERSTILVEIVAEGIAYEPG 722

pseudopunctipennis YAPVNEQESLDLALSKFHNKKATVCSAKRDPINLHGEMNATERSTIRVEIFAEGMGYEPG 237

sinensis YAPVQEHESLDRALSKFHNKKSTECSVKRNPINLHCEMNGTERSTILVEIVAEGIDYEPG 968

gambiae YAPVSENEPLDRALSKFHNKKSMECSVKRNPINLHCEMNGTERSTILVEIMAEGIDYEPG 717

dirus YAPVSEYESLDRALSKFHNKKSMECSVKRNPINLHCEMNGTERSTILVEIMAEGIDYEPG 612

stephensi YAPVAEYESLDRALSKFHNKKSMECSVKRNPINLHCEMNGTERSTILVEIMAEGIDYEPG 858

Drosophila DHVGIFPANRTELVDGLLNRLVGVDNPDEVLQLQLLKEKQTSNGIFKCWEPHDKIPPDTL 769

Culex DHVGIFPANRAEIVDGIIKRLTGVTDPDETLQLQVLKEKQTQNGVYKSWEQHERIPICSL 293

darlingi DHVGIFPANRKDIVDGIIERLVGVTNPDEILQLQLLKEKQTANGVYKAWEPHERIPSCSL 1151

aquasalis ------------------------------------------------------------ 0

Aedes DHVGIFPANRPEIVDGIIERLSGVDNPDEILQLQVLKEKQTQNGVYKSWEQHERLPICSL 782

pseudopunctipennis DHVGIFPANRKEIVDGIIERLAGVNDPDEILQLQVLKEKQTQNGVYKSWEPHERLPVCSL 297

sinensis DHVGIFPANRKEIVDGIIERLTGVNDPDEMLQLQILKEKQTQNGVYKSWEPHERLPVCSL 1028

gambiae DHVGIFPANRKEIVDGIIERLTGVNDPDEELQLQVLKEKQTQNGVYKSWEPHERLPVCSL 777

dirus DHVGIFPANRKEIVDGIIERLSGVNDPDEMLQLQVLKEKQTQNGVYKSWEPHERLPVCAL 672

stephensi DHVGIFPANRKEIVDGIIERLTGVNDPDEMLQLQVLKEKQTQNGVYKSWEPHERLPVCTL 918

Drosophila RNLLARFFDLTTPPSRQLLTLLAGFCEDTADKERLELLVNDSSAYEDWRHWRLPHLLDVL 829

Culex RTLLSRFLDITTPPSRQLLTFLASCCQEKEDEERLTMLANEPSVYEDWRYWKLPHLLEVL 353

darlingi RTLLTRYLDITTPPTRQLLTYLATCCSEKSDEERLTMLANESSVYEDWRHWKLPHLLEVL 1211

aquasalis -----------------------------------------------------------L 1

Aedes RTLLSRFLDITTPPSRQLLTFLATCCDDKKDEERLLMLANESSVYEDWRYWKLPHLLEVL 842

pseudopunctipennis RTMLTRFLDITTPPTRQLLTYLASCCSDKADEERLLMLANESSVYEDWRYWKLPHLLEVL 357

sinensis RTLLTRFLDITTPPTRQLLTYLASCCGDKADEERLLMLANESSVYEDWRYWKLPHLLEVL 1088

gambiae RTLLTRFMDITTPPTRQLLTYLASCCSDKADEERLLMLANESSVYEDWRYWKLPHLLEVL 837

dirus RTLLTRFLDITTPPTRQLLTYLASCCGDKADEERLLMLANESSVYEDWRYWKLPHLLEVL 732

stephensi RTLLTRFLDITTPPTRQLLTYLASCCGDKADEERLLMLANESSVYEDWRYWKLPHLLEVL 978

*

Drosophila EEFPSCRPPAPLLLAQLTPLQP---RFYSISSSPRRVSDEIH------------------ 868

Culex EEFPSCKPPATVFVAKLNLSSLGSTRFLSLRATPNSDVNLFRRPRSLPQLGQQLTLFTPS 413

darlingi EEFPSCHPPAAVLVAQLSPLQP---RFYSISSSPRKYSNEIH------------------ 1250

aquasalis EEFPSCHPPAAVLVAQLSPLQP---RFYSISSSPRKYPNEIH------------------ 40

Aedes EEFPSCKPPATVLVAQLNALQP---RFYSISSSPRKYSNEIH------------------ 881

pseudopunctipennis EEFPSCHPPAAVIVAQLNALQP---RFYSISSSPRKYLNEIH------------------ 396

sinensis EEFPSCRPPAAVLVAQLNALQP---RFYSISSSPRQYPNEIH------------------ 1127

gambiae EEFPSCRPPAAVFVAQLNALQP---RFYSISSSPRKYSKEIH------------------ 876

dirus EEFPSCHPPAAVFVAQLNALQP---RFYSISSSPRKYSNEIH------------------ 771

stephensi EEFPSCRPPAAVFVAQLNALQP---RFYSISSSPRKYSNEIH------------------ 1017

******:*** :::*:*. . ** *: ::*. . ::

Drosophila --------------------------------------------------LTVAIVKYRC 878

Culex TSGIRKPPTSSKKISAEALSKLGPFPATSMESPAMPNVESSWSTAVTTAAVTVELVSSFR 473

darlingi --------------------------------------------------LTVAIVKYRA 1260

aquasalis --------------------------------------------------LTVAIVKYRV 50

Aedes --------------------------------------------------LTVAIVSYRA 891

pseudopunctipennis --------------------------------------------------LTVAVVTYRA 406

sinensis --------------------------------------------------LTVAIVTYRA 1137

gambiae --------------------------------------------------LTVAIVTYRA 886

dirus --------------------------------------------------LTVAIVTYRA 781

stephensi --------------------------------------------------LTVAIVTYRA 1027

:** :*.

Drosophila EDGQGDERYGVCSNYLSGLRADDELFMFVRSALGFHLPSDRSRPIILIGPGTGIAPFRSF 938

Culex RHGEGAEHYGVCSNYLANMVDNEKIYLFVRSASSFHMSKDPSRPVILIGPGTGIAPFRSF 533

darlingi QDGKGAEHYGVTSNYLADLDPEERLLLFVRSAPSFHMPKDPTKPIILIGPGTGIAPFRSF 1320

aquasalis QDGSGAEHYGVTSKYLVDLDPQEGVLLFVRSAPSFHMPKDPTKPIILIGPGTGIAPFRSF 110

Aedes EDGEGAEHYGVCSNYLANLDGEDKMFLFVRSASSFHMSKDPSRPVILIGPGTGIAPFRSF 951

pseudopunctipennis EDGEGAEHYGVCSNYLANLQADDKIFLFVRSAPSFHMSTDPTKPVILIGPGTGIAPFRSF 466

sinensis EDGEGAEHYGVCSNYLANLQPEDRIFLFVRSAPSFHMSKDPTKPVILIGPGTGIAPFRSF 1197

gambiae EDGEGAEHYGVCSNYLANLQPDDKIFLFVRSAPSFHMSKDPTRPVILIGPGTGIAPFRSF 946

dirus EDGEGAEHYGVCSNYLANLQPDDKIYLFVRSAPSFHMSKDPTKPVILIGPGTGIAPFRSF 841

stephensi EDGEGAEHYGVCSNYLANLQSDDKIYLFVRSAPSFHMSKDRTKPVILIGPGTGIAPFRSF 1087

..*.* *:*** *:** .: :: : :***** .**: .* ::*:***************

Drosophila WQEFQVLSDLDPTAKLPKMWLFFGCRNRDVDLYAEEKAELQKDQILDRVFLALSREQAIP 998

Culex WQEWDTLKTELPDVKLPKVWLFFGCRTKKVDLYRDEKEEMVKKGILDRVFLALSREENVP 593

darlingi WQEWHYRKM-EIGEQIPKVWLFFGSRTRSLDLYRDEKEEMLQQAILDRVFLALSREKDIP 1379

aquasalis WREWLYRKL-EVGEHIPKVWLFFGSRTRSLDLYRDEKEDMVQQGILDRVFLALSREKVIP 169

Aedes WQEWSTIKQALPESEIPKVWLFFGCRTKKVDLYRDEKEEMVQHGILDRVFLALSREENVP 1011

pseudopunctipennis WQEWDHIKTEMVDSKIPKVWLFFGCRSREA------------------------------ 496

sinensis WQEWDHIKTEMVDSKIPKVWLFFGCRTKNVDLYRDEKAEMVKKGVLDRVFLALSREENIP 1257

gambiae WQEWDHIKSEMVDCKIPKVWLFFGCRTKNVDLYRDEKEEMVQKGVLDRVFLALSREENIP 1006

dirus WQEWDHIKTEMVDCKIPKVWLFFGCRTKNVDLYRDEKQEMVQNGVLDRVFLALSREENIP 901

stephensi WQEWDHIKTEMVDCKIPKVWLFFGCRTKNVDLYRDEKEEMVQHGVLDRVFLALSREENIP 1147

*:*: . .:**:*****.*.:.

Drosophila KTYVQDLIEQEFDSLYQLIVQERGHIYVCGDVTMAEHVYQTIRKCIAGKEQKSEAEVETF 1058

Culex KTYVQDLALTEADSIFDLIWNEKAHIYVCGDVTMAEHVYQTLRKILAGKLTKTESEMEKY 653

darlingi KTYVQDLALKEADAIAPLILREKGHVYVCGDVTMAEHVYQTLRKILATHENKTETEMEKY 1439

aquasalis KAYVQDLALKEADAIAPLILREKGHVYVCGDVTMAEHVYQTLRKILATYENKTETEMEKY 229

Aedes KTYVQDLALKESDSIFELIWNEKAHIYVCGDVTMAEHVYQTLRRILATKLNKTESEMEKY 1071

pseudopunctipennis ------------------------------------------------------------ 496

sinensis KTYVQDLALKEAESICQLIMQEKAHIYVCGDVTMAEHVYQTLRKILATRENRTETEMEKY 1317

gambiae KTYVQDLALKEADSISELILQEKAHIYVCGDVTMAEHVYQTLRKILATHENRTESEMEKY 1066

dirus KTYVQDLALKEADSISELIMQEKAHIYVCGDVTMAEHVYQTLRKILATREKRTETEMEKY 961

stephensi KTYVQDLALKEAESISELIMQEKGHIYVCGDVTMAEHVYQTLRKILATREKRTETEMEKY 1207

Drosophila LLTLRDESRYHEDIFGITLRTAEIHTKSRATARIRMASQP-------------------- 1098

Culex MLSLRIDRRSIDSYVVWSRTKVSSLITECAIVDARASSKNLRRSCTYMRGTTSLRWTCSG 713

darlingi MLTLRDENRYHEDIFGITLRTAEIHNKSRANARIRMGQSCSS------------------ 1481

aquasalis MLTLR------------------------------------------------------- 234

Aedes MLSLRDENRYHEDIFGITLRTAEVHNKSRATARIRMASQPS------------------- 1112

pseudopunctipennis ------------------------------------------------------------ 496

sinensis MLTLRDENRYHEDIFGITLRTAEIHNKSRCGLYIAICLTKGIATEHEQETGRCIASLPRK 1377

gambiae MLTLRDENRYHEDIFGITLRTAEIHNKSRATARIRMASQP-------------------- 1106

dirus MLSLRDENRYHEDIFGITLRTAEIHNKSRATARIRMASQP-------------------- 1001

stephensi MLTLRDENRYHEDIFGITLRTAEIHNKSRATARIRMASQP-------------------- 1247

Drosophila ------------------------------------------------------------ 1098

Culex FCAELT------------------------------------------------------ 719

darlingi ------------------------------------------------------------ 1481

aquasalis ------------------------------------------------------------ 234

Aedes ------------------------------------------------------------ 1112

pseudopunctipennis ------------------------------------------------------------ 496

sinensis YKPSVHVSYGQLLLTCRICMYGALKFPAIDRHPRPSSEVMVNDHHSNLENGKQHEPQSHV 1437

gambiae ------------------------------------------------------------ 1106

dirus ------------------------------------------------------------ 1001

stephensi ------------------------------------------------------------ 1247

Drosophila ------------------------------------------------------------ 1098

Culex ------------------------------------------------------------ 719

darlingi ------------------------------------------------------------ 1481

aquasalis ------------------------------------------------------------ 234

Aedes ------------------------------------------------------------ 1112

pseudopunctipennis ------------------------------------------------------------ 496

sinensis SFDQTCQIDVILRRLGGPIIPILHADAQNAASRSGPPELEMHNGQQIKQVMLPTSFTSRN 1497

gambiae ------------------------------------------------------------ 1106

dirus ------------------------------------------------------------ 1001

stephensi ------------------------------------------------------------ 1247

Drosophila ------------------------------------------------ 1098

Culex ------------------------------------------------ 719

darlingi ------------------------------------------------ 1481

aquasalis ------------------------------------------------ 234

Aedes ------------------------------------------------ 1112

pseudopunctipennis ------------------------------------------------ 496

sinensis GNRNDNSACVSLRVSDSLTNTPSGTGEGRYIVKQGKQKTESTVRHLFW 1545

gambiae ------------------------------------------------ 1106

dirus ------------------------------------------------ 1001

stephensi ------------------------------------------------ 1247
