# Supplementary material for: Quantitative real-time PCR analysis of Anopheles dirus TEP1 and NOS during Plasmodium berghei infection, using three reference genes
Source: PeerJ. 2017 Jul 26;5:e3577. doi: 10.7717/peerj.3577 (PMC5533154; doi:10.7717/peerj.3577)
Supplement: File S4 [file peerj-05-3577-s004.docx]

>Anopheles dirus thioester-containing protein 1 (TEP1) putative complete cds (2733 bp)

ATGACACGGTTTCTGGTGAAGTGCAGTGATCAAATGGGATTCTTCGTATACTACGTGCTTTCGAAAGGCA

ACATCATTGATTCGGGTTACATCAGACCGAATAAGCAGACCAGCTATCTGCTCCAGTTGAACGCCACAGA

AAAAATGATACCGAAGTCGAAAATCGTTGTGGCCACCGTTACGAAGAATATAGTGGTATATGATTTCGTT

GATCTCAACTTTGACGAATTCCGTAACAATTTTGAATTAAATATCGATGAGGACGAGATTAAACCCGGCC

GGCAGATCGAACTGAGCATGCGTGGTCGACCAGGATCGTGCGTTGGATTAGCCGCGTACGATGCGAGTTT

GCTGGCCTATGGCAAGCACCATGATCTGTATTGGGATGACTTCGTGCAAGTTTTCAACGGTTTTCATAAT

ATCGACGATAATGAGTTTGATAAGATTCACAGTATGGGACTGTTTGCTAGAACCTTGGATGATATCATTT

TTGACGGTGCGAACGACAAATCCGCGCGTGCTGGTTCACAGTCCAACAAAGCGCCCACGAAGCTAGTTGA

ATTTCGAACGAACTTTGCGGAATCGTTTTTGTGGAAGAACGTGACCATCGGAAGACAGGGTACGCGCAGT

TTGATCGAGGTCGTTCCGGATACAACCACGTCCTGGTATCTGAAGGGATTCTCCATCGATCCAACCTACG

GGTTGGGCATCATAAAAAAGCCGATCGTGTTCAAAACGGTGCAGCCATTTTATATCGTTGAGAACTTGCC

GTATTCCATCAAGCGGGGGGAAGCGGTCGTGTTGCAGTTTACACTCTTCAACAGTCTCGGTGCAGAGTAT

ATCGCGGATGTGACGCTCTTAAATGTGGCCAACCAGACGGAGTTTGTCGGACGACCTTTGGAAGATGTGA

GCTACACGAAGTCCGTGTCCGTTCTACCCAACGTCGGTGTGCCGGTTTCATTTCTGGTGAAGGCCCGTAA

GCTCGGTGAGATGGTCGTCCGTGTTAAGGCATGGATAATGAACGGTCTGGAGACAGACGGGTTGGAGAAG

GTTATCCGCGTATTGCCAGAGAGCTTAGTGCAGCCAAGGATGCTGTCGCAGTTTTTCTGCTTCGATGAGT

ACAAAAACCAAACATTCCAGTTCAACTTAGACATCAACAAGCTGGCCGATAAAGGATCGGAGAAAATCAA

GTTCAGAGTTAATCCAAACTTGCTCTCATCGGTGATCGACAATTTGGATCATCTTCTCGCTGTGCCGACT

GGATGTGGGGAGCAGCATATGGTTAAGTTCGTACCGAACATCGTTGCGCTCGACTATCTAACCGCGGTCG

GTTCGAAGCAAAAGACGATAATTGACAAAGCTACTAATATGCTTCGCCAAGGATATCAGAACCAGATGCG

GTACCGCCAAGCAGACGGCTCCTTCGGTGTGTGGGGACATAGCGGGGGAAGTGTGTTCCTTACTGCGTTT

GTGGCAAAGTCCATGCAAACTGCATCCAAGTACATTGACGTTGATAAAGCGCTGGTTGAGAGGGCATACG

AGTGGCTCGCTTCCAAACAAAGCTTTTCGGGAAGGTTCGATGAGGTTGGTTCCGTTATCCACCAGGATAT

GCAAGGCGGTCTGCGGAATGGTGTAGCGCTGACATCGTACGTGTTGACGGCACTGCTGGAGCATCAGGGT

TCTAGGACTACATATGCGCAGCATATCCAGAAGGCCTTGCACTATCTGAATAATCAACTCGCTAGTATCA

ATGAACCTTACGACCTGTCCATAGCGACGTATGCGCTGATGCTGGGAAAACATATTAACAAGAACGAAGC

GCTGAAAAAACTTGTAGGACTGTCGACACCCTTAAATAACGGAACGGAACGGTATTGGAATACTGCAAAC

AAGATAGAGGCTACCGCGTATGCTTTGCTATCGTTTGTGGCCGCAGAGAAGTACATTGAGGGAATACCGA

TCATGCGTTGGCTGGTGGATCAGCGCTTCGTGACCGGAAGCTTCCCGCGGACGCAGGACACGTTTGTCGG

CCTTAAGGCACTGACGGAGCTGGCCGAGAAAATCTCCCCCTCGAGAAACGACTACAGCATCCAGGTGACG

ACGAAGAAGCTGCCTAAGCGCTATTTTATCAACTCTAAAGATGTTGTACCTTTGGAGGAGGAAAAATTTC

CACYTGATACGCGAAAAATGGAGGTGAACGTGGGGGGCCTTGGGTTTGGTTTGTTGGAAGTGATTTAMGA

GTACACGCTGAATCTGCARAACTTCTCGCATCGCTTTGACCTGAACCTGGAGAAGCAGGACACCGGTTCG

GATTATGCGTTGCAGCTTAAAGTGTGTACCAGCTACATTCCAAAACTGTCRAACGAGCGGTCGAACATGG

CGCTGGTCGAGGTGARCTTACCGAGCGGGTATGTCGCTGACCGGAATCCGATCAGCCAGCAGAYTACGAT

AAATCCGATTCAGAACATCGAACTTCGGTACGGTAGCACATCCATCGTAGCATACTATAACAACATGGGC

ACCGAGAAGAACTGTTTCACTGTGACGGCCTACAAACGTTTCAAGGTGGCCCTGATACGGCCGGCGTACG

TAGTTGTGTACGATTACTACAATGCTGATYTGAACGCCATCAAATTGTACGAAGTYTTYTCCCAGAAGAT

ATGCGATTTGTGCGAAGAGGGCAGCTGTCCTGACGAATGCTCCTTCTCCCCAATAGCTGCGGAGACTACG

TGA

>Anopheles dirus nitric oxide synthase putative complete cds (3006 bp)

ATGTTTGAAGCGATCTGCAATCRCATCAAATAYGCCACCA

ATAAAGGAAACCTCAGATCGGCCATCACAATTTTCCCACA

GCGAACGGACGGCAAGCACGACTACCGCATCTGGAACAAT

CAGATTATCTCGTACGCCGGCTATAAGAACGCAGACGGTA

AAATCATCGGTGATCCGGCCAATGTCGAGTTTACTGATTT

TTGCGTGAAGCTCGGCTGGAAGAGCAAACGGACGGAGTGG

GACATCCTGCCGCTGGTCGTGTCGGCGAACGGGCACGATC

CGGACTACTTTGACTACCCGCCGGAGCTGATCCTGGAGGT

GCCGCTCAGCCACCCCCAGTTCAAGTGGTTCGCCGAGCTG

AACCTGCGGTGGTACGCGGTGCCGATGGTGTCGTCGATGC

TGTTCGACTGCGGTGGCATCCAGTTCACGGCGACGGCCTT

CAGCGGCTGGTACATGTCGACCGAGATCGGCTGCCGCAAC

CTGTGCGACACCAACCGCCGAAACCTGCTCGAGCCAATCG

CAATCAAAATGGGGCTGGACACGCGAAACCCGACGTCACT

GTGGAAGGACAAGGCACTGGTGGAGATTAATATTGCCGTG

CTGCACTCGTACCAGAGCCGCAATATTACCATCGTCGATC

AYCACACCGCCAGCGAGAGCTTCATGAAGCACTTCGAGAA

CGAAACGAAGCTGAGGAACGGCTGTCCGGCCGACTGGATA

TGGATCGTGCCGCCSATGTCAGCCTCGGTAACGCCCGTCT

TCCACCAGGAGAWGGSCGTTTACTATCTGCGGSCCTCGTT

CGAGTATCAKGAAACGGCCMTGAAAACGCACATCTGGAAG

AAGGGACGAGATTCGGCGAAAAMCAAGAAACCCCGCCGAA

AGTTCAACTTCAAGCAAATAGCTAGAGCTGTCAAATTTAC

ATCGAAACTGTTCGGACGCGCCCTGTCCCGGCGGATTAAG

GCGACCGTGCTGTACSCGACGGAAACCGGACGCTCGGAGC

AGTACGCCCGTCAGCTCGTCGAACTGCTCGGCCATGCGTT

CAATGCACAGATCTACTGCATGTCGGATTACGACATCTCA

TCAATCGAACACGAGGCCCTGCTGCTGGTGGTCGCGTCCA

CCTTCGGCAACGGCGATCCACCGGAAAATGGCGAGCTCTT

CGCGCAGGATCTGTACGCGATGAAGCTCCACGAGAGCGGC

CACCATCAGGCGCACAGCGAGCTGACGATTGCCGCCTCGT

CGAAGTCGTTCATCAAGACGAACTCCCGGAGCGATCTGGG

CAAGTTTGGGCCGGCCGGCGCCCGCAAGATCGACCGGCTC

GATTCGCTGCGCGGTTCCACCACGGACACGCTGTCGGAGG

AGACGTTCGGTCCGCTGTCGAACGTCCGGTTCGCCGTGTT

CGCGCTCGGTTCGTCGGCCTACCCGAACTTCTGCGCCTTC

GGTAAGTACATCGACAACATCCTCGGCGAGCTGGGCGGCG

AGCGGCTGATGAAGATGGCGACCGGGGACGAGATCTGCGG

CCAGGAGCAGGCCTTCCGCAAGTGGGCCCCGGAGGTGTTC

AAGATCGCCTGCGAAACGTTCTGTCTCGATCCGGAGGAAA

CGCTCTCGGATGCGGCGTTTGCCCTGCAGAGCGAGCTGTC

GGAGAACACCGTGCGCTACGCGCCGGTCAGCGAGTACGAG

TCGCTCGACCGGGCGCTGTCCAAGTTCCACAACAAAAAGT

CGATGGAGTGCTCGGTGAAGCGGAACCCGATCAATCTGCA

CTGCGAGATGAACGGCACGGAGCGGTCGACGATTTTGGTT

GAGATTATGGCCGAAGGGATTGATTACGAACCAGGCGATC

ATGTGGGAATTTTCCCGGCAAATCGGAAGGAAATTGTCGA

CGGAATTATCGAACGTCTGAGCGGCGTGAATGATCCCGAC

GAGATGCTGCAGCTGCAGGTGCTGAAGGAGAAGCAGACAC

AGAATGGTGTCTACAAATCTTGGGAGCCTCACGAGAGGCT

GCCTGTTTGTGCGCTCCGCACACTGTTGACCCGGTTCCTG

GACATTACCACGCCACCGACCAGACAGCTGCTCACGTATC

TTGCTTCGTGCTGCGGTGATAAGGCGGATGAGGAACGTTT

GCTTATGTTGGCCAATGAGTCATCGGTGTACGAAGACTGG

CGCTACTGGAAGCTGCCCCACCTGCTCGAGGTGCTGGAAG

AGTTTCCATCGTGTCATCCTCCGGCGGCCGTCTTCGTCGC

CCAGCTGAACGCGCTACAGCCGCGCTTCTACTCGATCTCG

TCCTCCCCGAGAAAGTACTCGAACGAGATCCATCTCACGG

TGGCCATCGTCACGTACCGGGCCGAGGATGGCGAGGGTGC

CGAGCACTACGGCGTCTGCTCGAACTATCTGGCCAACCTG

CAGCCGGACGACAAGATCTATCTGTTCGTGCGCAGCGCAC

CGTCGTTCCACATGTCGAAGGACCCGACCAAGCCGGTCAT

CCTGATCGGTCCCGGCACCGGTATCGCACCGTTCCGCTCG

TTCTGGCAGGAGTGGGACCACATCAAGACGGAAATGGTTG

ACTGTAAGATTCCCAAGGTATGGCTCTTCTTCGGCTGTCG

TACGAAGAACGTGGACCTATACCGGGACGAGAAGCAGGAG

ATGGTCCAGAACGGTGTACTCGATCGGGTGTTCCTCGCAC

TGTCGCGCGAGGAAAACATCCCTAAGACGTACGTACAGGA

CCTCGCCCTGAAGGAGGCGGACTCGATCTCCGAGCTGATC

ATGCAGGAGAAAGCGCACATCTACGTCTGCGGCGACGTCA

CCATGGCCGAGCACGTGTACCAAACGCTGCGCAAGATACT

GGCCACGCGCGAAAAGCGCACGGAAACGGAAATGGAGAAG

TACATGCTGTCACTGCGGGACGAAAACCGCTACCACGAGG

ACATTTTCGGCATCACGCTGCGCACGGCCGAGATCCACAA

CAAGTCACGCGCCACCGCCCGCATTCGTATGGCATCGCAG

CCGTAA

>Anopheles dirus elongation factor 1-alpha (EF1) partial DNA sequence (1137 bp)

ATGGGTAAGGAGAAGACTCATATTAACATCGTCGTCATCGGACACGTCGATTCCGGCAAGTCGACCACCACCGGTCATTTGATCTACAAATGCGGCGGTATCGACAAGCGTACGATCGAGAAGTTCGAGAAGGAGGCCCAGGAGATGGGCAAGGGCTCGTTCAAGTACGCCTGGGTGCTGGACAAGCTGAAGGCCGAGCGTGAGCGTGGTATCACCATCGATATCGCGCTGTGGAAGTTCGAAACCTCCAAGTACTACGTCACCATCATCGACGCCCCCGGACATCGTGATTTCATCAAGAACATGATCACGGGAACGTCGCAGGCCGATTGTGCCGTGCTGATCGTGGCTGCCGGTACTGGTGAGTTCGAGGCCGGTATCTCCAAGAACGGCCAGACCCGTGAGCACGCGCTGCTCGCCTTCACGCTCGGTGTGAAGCAGCTGATTGTTGGTGTGAACAAGATGGACTCGACCGAGCCGCCGTACAACGAGGCCCGTTTCGAGGAAATCAAGAAGGAGGTGTCGTCGTACATCAAGAAGATCGGTTACAACCCGGCCGCCGTCGCGTTCGTCCCGATCTCCGGATGGCACGGAGACAACATGCTGGAACCCTCCACCAAGATGCCGTGGTTCAAGGGATGGGCCATCGAGCGCAAGGAGGGTAAGGCTGACGGCAAGTGCCTGATTGAGGCCCTGGACGCCATCCTGCCGCCGTCCCGTCCCACCGACAAGCCGCTGCGTCTGCCCCTGCAGGACGTGTACAAAATCGGCGGTATCGGAACAGTCCCGGTCGGTCGTGTGGAAACCGGTGTGCTGAAGCCCGGTACCGTGGTCGTCTTCGCCCCGGTCAACCTCACCACTGAGGTCAAGTCGGTGGAGATGCACCACGAAGCGCTGCAGGAGGCCGTGCCCGGCGACAACGTCGGTTTCAACGTGAAGAACGTGTCGGTGAAGGAACTGCGTCGCGGTTACGTCGCCGGTGACTCGAAGAACGCGCCGCCCAAGGGTGCCGCCGACTTCACCGCCCAGGTCATCGTGCTGAACCACCCGGGACAGATCAGCAACGGCTACACGCCGGTGCTCGATTGCCACACCGCCCACATTGCGTGCAAGTTCTCCGAGATCAAGGAGAAGGTC

>Anopheles dirus actin-1 (Act) partial DNA sequence (683 bp)

ATGGTCGGCATGGGRCAGAAGGACTCGTACGTCGGCGACGAGGCCCAGAGCAAGCGTGGTATCCTGACGCTGAAGTACCCGATCGAGCACGGCATCATCACGAACTGGGATGATATGGAGAAGATCTGGCACCACACGTTCTACAACGAGCTGCGTGTGGCCCCGGAGGAGCACCCGGTCCTGCTGACTGAGGCCCCGCTGAACCCGAAGGCTAACCGCGAGAAGATGACBCAGATCATGTTCGAGACGTTCAACTCGCCGGCBATGTACGTCGCCATCCAGGCCGTGCTGTCGCTGTACGCCTCCGGTCGTACCACCGGTATTGTGCTGGACTCCGGTGATGGTGTCTCGCACACCGTCCCGATCTACGAAGGTTACGCGCTGCCVCATGCCATCCTCCGTCTGGATCTGGCTGGTCGCGATCTGACCGACTACCTGATGAAGATCCTGACCGAGCGCGGCTACTCGTTCACCACCACCGCTGAGCGTGAAATCGTGCGTGACATCAAGGAGAAGCTGTGCTACGTTGCGCTGGACTTCGAGCAGGAGATGGCCACCGCCGCCGCCTCCACCTCGCTGGAGAAGHCGTACGAGCTGCCBGACGGACAGGTCATCACCATCGGCAACGAGCGCTTCCGCTGCCCGGAGGCTCTGTTCCAGCCTTCCTTCCTGGGTATGGAATC
